# Oleoylethanolamide treatment reduces neurobehavioral deficits and brain pathology in a mouse model of Gulf War Illness

**DOI:** 10.1038/s41598-018-31242-7

**Published:** 2018-08-27

**Authors:** Utsav Joshi, James E. Evans, Ross Joseph, Tanja Emmerich, Nicole Saltiel, Carlyn Lungmus, Sarah Oberlin, Heather Langlois, Joseph Ojo, Benoit Mouzon, Daniel Paris, Michael Mullan, Chao Jin, Nancy Klimas, Kimberly Sullivan, Fiona Crawford, Laila Abdullah

**Affiliations:** 1 Roskamp Institute, 2040 Whitfield Ave, Sarasota, FL 34243 USA; 2 Open University, Milton Keynes, United Kingdom; 3 James A. Haley Veterans’ Hospital, Tampa, Florida USA; 4 NOVA Southeastern University, Ft. Lauderdale, FL USA; 5 Miami VAMC, Miami, FL USA; 6 Boston University School of Public Health, Boston, MA USA

**Keywords:** Lipid-storage diseases, Neuroimmunology

## Abstract

There are nearly 250,000 Gulf War (GW) veterans who suffer from Gulf War Illness (GWI), a multi-symptom condition that remains untreatable. The main objective was to determine if targeting peroxisomal function could be of therapeutic value in GWI. We performed a pilot study that showed accumulation of very long chain fatty acids (VLCFA), which are metabolized in peroxisomes, in plasma from veterans with GWI. We then examined if targeting peroxisomal β-oxidation with oleoylethanolamide (OEA) restores these lipids to the normal levels and mitigates neuroinflammation and neurobehavioral deficits in a well-established mouse model of GWI. In GWI mice, treatment with OEA corresponded with cognitive benefits and reduced fatigue and disinhibition-like behavior in GWI mice. Biochemical and molecular analysis of the brain tissue showed reduced astroglia and microglia staining, decreased levels of chemokines and cytokines, and decreased NFκB phosphorylation. Treatment with OEA reduced accumulation of peroxisome specific VLCFA in the brains of GWI mice. These studies further support the translational value of targeting peroxisomes. We expect that OEA may be a potential therapy for treating neurobehavioral symptoms and the underlying lipid dysfunction and neuroinflammation associated with GWI. Oleoylethanolamide is available as a dietary supplement, making it appealing for human translational studies.

## Introduction

Gulf War Illness (GWI) affects nearly a third of the US military personnel deployed in the 1991 Gulf War (GW), who continue to experience multiple symptoms including chronic pain, fatigue, and memory impairment^1^. To date, GWI remains difficult to understand but is thought to have a central nervous system (CNS) component characterized by damage to the brain regions involved in storing and processing memory and the axonal fibers transmitting sensations of pain and fatigue^1,2^. Animal studies suggest a pathogenic mechanism involving activation of the brain astroglia and microglia cells in promoting neuroinflammation that corresponds with metabolic disturbances following exposure to GW chemicals^1^. It is therefore important to explore treatment strategies that are geared toward targeting biological disturbances in the brain to determine if such approaches may alleviate the symptoms and pathology of GWI.

Evidence for CNS involvement in GWI also comes from animal modeling studies that used chemicals that were widely used by military personnel during the GW conflict^3^. These studies showed neurobehavioral deficits such as depression and cognitive problems corresponding with chronic brain astroglia and microglia activation, appearing at chronic post-exposure periods^3–9^. Extensive characterization of these GWI mouse models using proteomic, lipidomics, and metabolomics technologies suggests the presence of inflammation and impaired lipid metabolism^6,8^. Further analyses of these GWI mouse models at early post-exposure time-points (ranging from 1- to 5-months) showed changes in omega-3 docosahexaenoic acid (DHA) and ether-containing phospholipids (PL), as well as very long chain-fatty acid (VLCFA) containing sphingomyelin (SM) species in the brains of GW agent-exposed mice compared to control mice^5,10^. Many of these lipids decline with aging and chronicity of the post-exposure time-points^8^. These lipid changes are also detected in the blood of veterans with GWI^11^. Since metabolism of VLCFA and synthesis of DHA and ether-containing PL is dependent upon normal peroxisomal function^12^, these studies suggest a role of altered peroxisomal function in the chronic pathology of GWI.

Peroxisomes are the site of α-oxidation of 3-methyl branched-chain fatty acids (BCFA) and β-oxidation of VLCFA^13–15^. Incomplete β-oxidation of VLCFA may lead to an overproduction of reactive oxygen species (ROS) or inadequate processing of ROS by the resident catalase in the peroxisomes, which may trigger oxidative stress and upregulate inflammatory responses^16–18^. Recent studies have also shown an increased expression of genes related to oxidative stress and inflammation in a rat model of GWI^19^. Support for a role of inflammation in GWI also comes from clinical studies showing increases in pro-inflammatory cytokines, such as Tumor Necrosis Factor (TNF)-α, Interferon-gamma (IFN-γ), Interleukin 2 (IL-2) and IL-1β, on immune cells and/or in plasma from veterans with GWI compared to healthy veterans^20–23^. Collectively, these studies suggest that disturbances in peroxisomal β-oxidation may be associated with the oxidative stress and inflammation observed in GWI. Thus, targeting peroxisomes may be useful for treating GWI.

We, therefore, hypothesize that GW chemicals may have disturbed peroxisome function, which could impair β-oxidation or aberrantly increase VLCFA synthesis. As such, targeting peroxisome function may be beneficial for treating GWI. Peroxisome proliferator-activated receptor alpha (PPARα) is a nuclear hormone receptor which stimulates peroxisome proliferation, thereby increasing peroxisomal function and regulating lipid homeostasis^24–28^. Furthermore, PPARα agonists are also shown to reduce inflammation through genetic regulation of nuclear factor kappa B (NFκB) in various CNS disorders^29–32^. We, therefore, expect that Oleoylethanolamide (OEA), being a PPARα agonist, may be of use in treating the underlying lipid dysfunction associated with GWI in a mouse model. Thus, the main goal of this study is to examine the fatty acid (FA) profiles associated with peroxisome function in blood from veterans with GWI and to test whether OEA treatment alters peroxisomal lipid metabolism and improves the chronic neurobehavioral deficits and glial activation in a mouse model of GWI.

## Results

### Peroxisome-associated VLCFA and BCFA are altered in plasma from veterans with GWI

**Figure 1:**
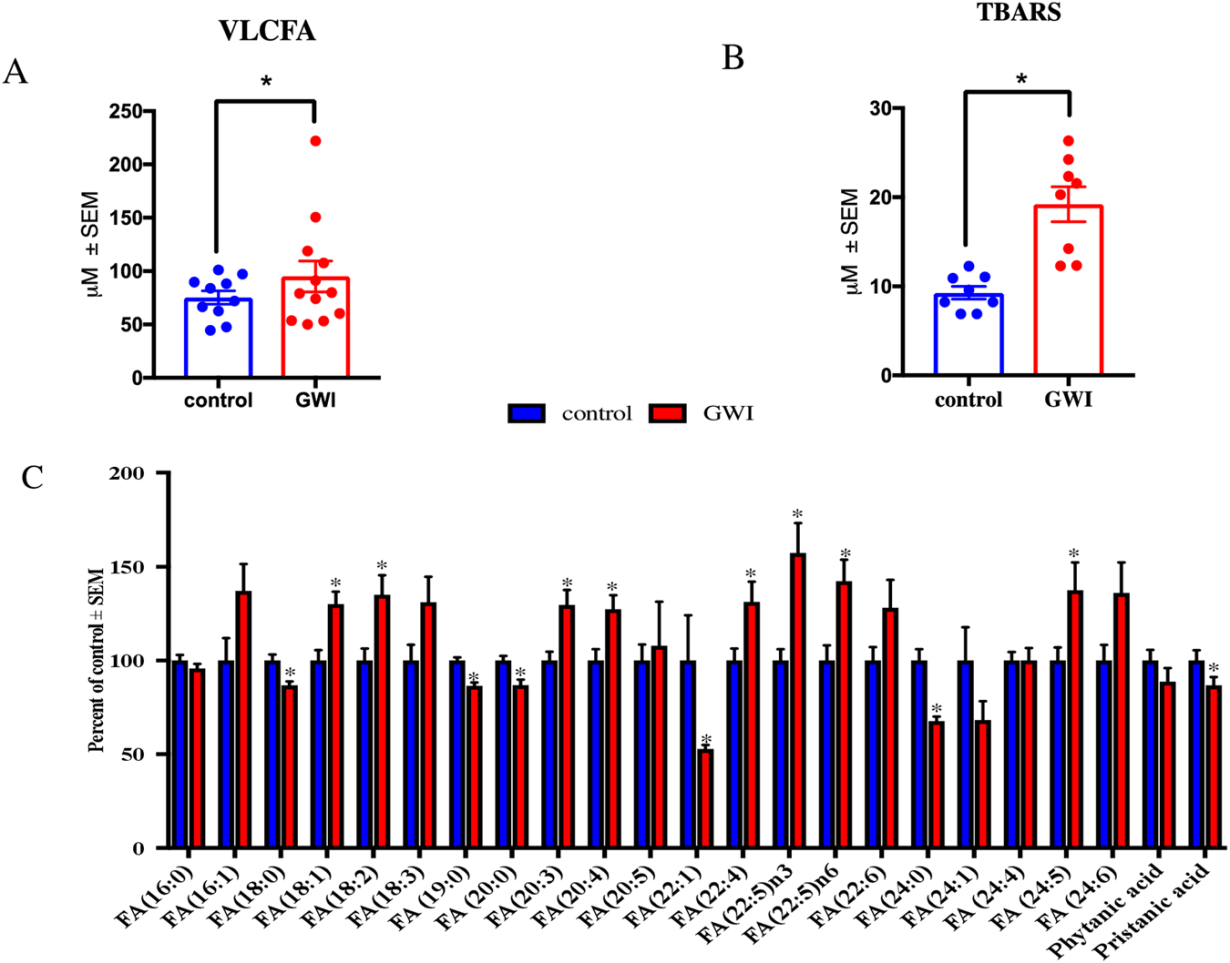
Plasma FFA profiles in veterans with GWI compared to GW control veterans. Mean ± SEM (n = 10 for controls and n = 12 for GWI). (**A**) When all VLCFA (C 22), were combined as one category, there was an overall increase in GWI compared to control GW veterans. (**B**) Quantification of lipid peroxidation product TBARS (n = 8 per group for these analyses only) in plasma showed that levels were significantly elevated in GWI compared to control veterans. (**C**) Profiling of FFA showed that several saturated (no double-bonds) and monounsaturated fatty acids (one double-bond) were decreased in the blood of veterans with GWI compared to controls. Pristanic acid was decreased in GWI compared to controls. The remaining long-chain fatty acids (14–21 carbons) and VLCFA species particularly omega-3 and omega-6 species, were significantly elevated in veterans with GWI compared to controls. *p ≤ 0.05.

In a pilot cross-sectional study of age- and gender-matched healthy GW veterans (n = 10) and those with GWI (n = 12), we examined pristanic acid (a BCFA), VLCFAs, and several free fatty acids (FFA). Figure 1A shows that levels of VLCFA were significantly increased (p < 0.05) in plasma from veterans with GWI compared to control GW veterans. Plasma levels of malondialdehyde (MDA) were measured using a Thiobarbituric Acid Reactive Substance (TBARS) formed during the reaction of MDA with thiobarbituric acid. Levels of MDA were two-fold higher in GWI compared to control GW veterans (p < 0.05, Fig. 1B). Pristanic acid levels were decreased in GWI compared to controls (p = 0.05, Fig. 1C). An increase in several omega-3 (FA18:3, FA20:5n3, FA 22:5n3) and omega-6 (FA18:2, FA20:4, FA22:4 and FA 22:5) FFA was observed in plasma from GWI compared to control subjects (Fig. 1C). An effect size (d) ≥ 1.0 was detected for the differences between control and GWI subjects for VLCFA, pristanic acid and TBARS.

### OEA treatment improves neurobehavioral deficits in a GWI mouse model

**Figure 2:**
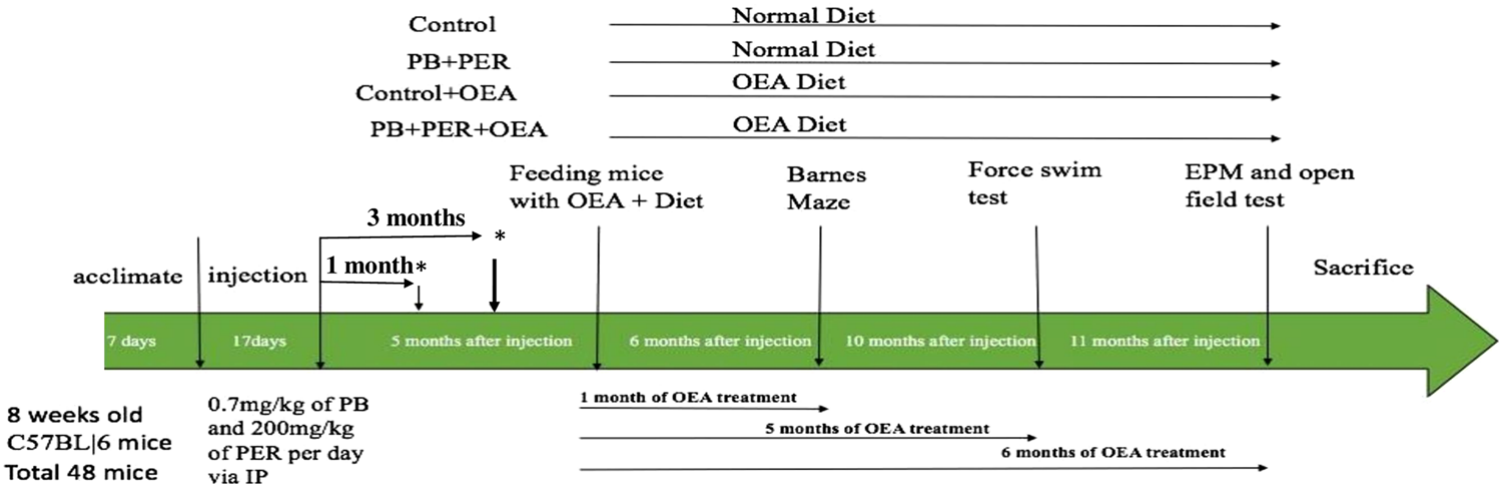
The experimental paradigm. The experimental timeline details the timing of GW chemical administration, treatment with OEA, behavioral testing, and tissue collection.

**Figure 3:**
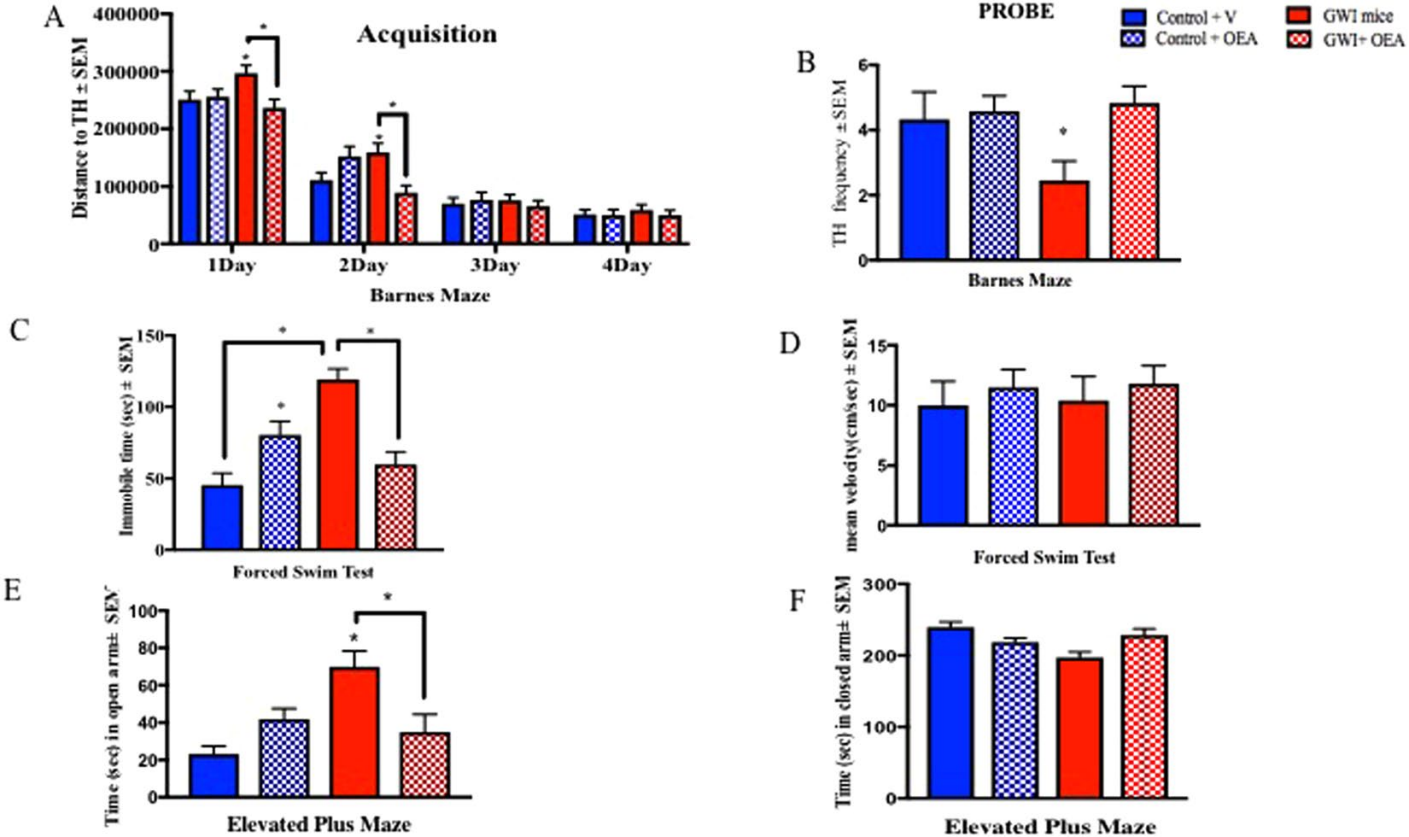
OEA treatment improves cognitive function and reduces fatigue and disinhibition type behavior in GWI mice. Mean ± SEM of control (n = 12), GWI mice (n = 11), control + OEA (n = 12) and GWI + OEA (n = 12). (**A**) One month after OEA treatment (6-months post-exposure to GW chemicals), acquisition trials were conducted to train mice to escape into the TH. For GWI mice treated with OEA, total distance to the TH was smallest, particularly on days 1 and 2, indicative of learning. As expected, GWI mice on normal chow had the worst performance with the largest total distance to the TH. Control mice treated with OEA had a higher latency to find the TH compared to control mice on the normal diet on day 2 only. (**B**) For the probe trials, conducted at 2-months post-treatment (7-months post-exposure), OEA treated GWI mice had a similar frequency of visits to the TH as control mice on normal chow and on OEA but a higher frequency of visits than GWI mice. (**C**) The immobile time was significantly increased in GWI mice compared to controls and was reduced in OEA treated GWI mice. (**D**) There was no change in the average speed among the different groups. (**E**) An examination of the time in the open arms of the EPM showed an increase of disinhibition in GWI mice on normal chow compared to both control groups and OEA treated GWI mice. (**F**) Total time in the closed arms was decreased in GWI mice compared with the control groups and OEA treated GWI mice. *p ≤ 0.05.

Based on the suggestion of possible peroxisomal β-oxidation dysfunction observed in blood samples from GWI subjects described above, we tested whether targeting peroxisomal β-oxidation with OEA would improve the chronic learning and memory deficits observed in a well-characterized mouse model of GWI^6–8,33^. A timeline of the study procedures is provided in Fig. 2. As previously described, this mouse model is exposed to two GW chemicals: an anti-nerve agent pyridostigmine bromide (PB) and a pesticide permethrin (PER)^8,33^. After GW chemical exposure, mice underwent testing with the Forced Swim Test (FST) at 1- and 3-months post-exposure. Control and GWI mice were divided into groups that either received oral OEA treatment in diet (administered orally in chow at 10 mg/kg daily) or the normal diet for a total of six months. The Barnes Maze test was used to assess learning in control and GWI mice after 1-month of oral OEA treatment (6-months post-exposure to GW chemicals) and long-term memory after 2-months of treatment (7-months post-exposure). Learning was assessed during the acquisition trials in which mice were allowed to freely explore the maze for 3 min and escape into the target box through one of the holes in the platform. On days 1 and 2, GWI mice traveled a longer distance to reach the target hole (TH) than control mice. However, GWI mice treated with OEA had the best performance, as demonstrated by having the shortest travel time when compared to control mice treated with OEA as well as compared to control and GWI mice on the normal chow diet (p < 0.05, Fig. 3A). During the probe trial at 2-months post-treatment, OEA treated GWI mice had a higher frequency of visiting the TH compared to GWI mice on a normal diet (p < 0.05, Fig. 3B). An effect size (f) = 0.4 was noted for these comparisons.

To test whether OEA corrected fatigue-like behavior in GWI mice, we performed the FST at 5-months post-treatment. While this test can be used for assessing depression, it is also helpful in quantifying fatigue by examining immobility, particularly when mice are repeatedly introduced to the FST apparatus^34,35^, as is the case here, where all mice were subjected to the FST at 1- and 3-months post-exposure (Supplementary Figure 1A). We also observed that at 3-months post-exposure, immobility was similar between control and GWI mice for the first 2 min (Supplementary Figure 1B), but immobility increased in GWI mice with time, further suggesting that fatigue emerges with some time delay. At 5-months post-treatment (10-months post-exposure), GWI mice treated with OEA were less immobile than GWI mice on the control diet but mean velocity was not affected between the groups (p < 0.05, Fig. 3C,D, f > 1.0). However, OEA treated control mice were more immobile than control mice on normal diet (Fig. 3C). Anxiety/disinhibition was tested at 6-months post-treatment using the elevated plus maze (EPM) test, in which time spent in the closed arm indicates increased anxiety, whereas time spent in the open arms is indicative of disinhibition. Relative to controls, GWI mice spent more time in the open arms and an influence of OEA treatment was observed whereby time in open arms was reduced in OEA treated GWI mice (p = 0.001, Fig. 3E, f = 0.59). Time in the closed arms was reduced in GWI mice and was restored by OEA to similar levels as those observed in control mice on the normal diet (Fig. 3F) with no differences in the velocity between any of the treatment groups (Supplemental Fig. 2). Control mice on the OEA containing diet exhibited disinhibition when compared to control mice on the normal diet, but no anxiety-like behavior was observed. We used the open field test (OFT) to assess locomotor function, and found no differences between any of the groups for spending time in the center zone or at the perimeter of the circular platform of the OFT, suggesting no influence of OEA on locomotor function (p > 0.05, Supplemental Fig. 3).

### Elevated levels of VLCFA in the brains of GWI mice were reduced by OEA treatment

**Figure 4:**
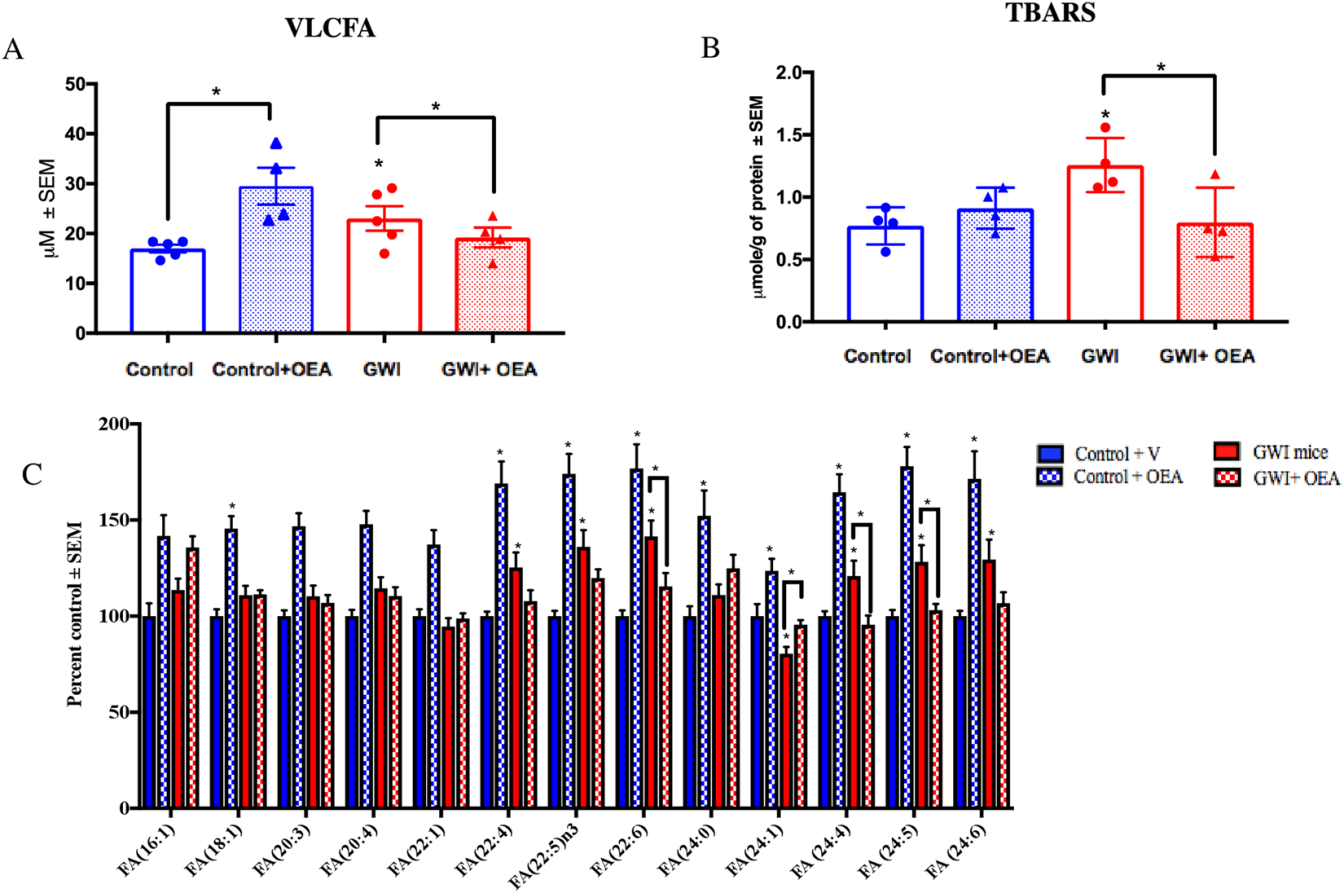
Brain lipid profiles show that OEA significantly decreases the VLCFA in the brains of GWI mice. Mean ± SEM expressed as percent control of mice on normal chow (n = 4/5 per group). (**A**) Levels of VLCFA were increased in the brains of GWI mice compared to control mice. OEA treated GWI mice had similar levels as in control mice. However, control mice treated with OEA had significantly higher levels of VLCFA compared to control mice that received normal diet. (**B**) Quantification of lipid peroxidation product TBARS showed that levels were significantly elevated in GWI compared to control mice on normal chow but were lower in OEA treated GWI mice compared to all other groups. (**C**) Compared to controls, GWI mice had higher levels of several major omega-6 and omega-3 FA (FA20:4, FA22:4. FA22:5, FA24:5 and FA24:6). Treatment with OEA decreased the levels of these FA in GWI mice. *p ≤ 0.05.

Levels of VLCFA were significantly elevated in the brains of GWI compared to control mice. Compared to GWI mice on the normal diet, those treated with OEA had lower levels of VLCFA and were similar to control mice on the normal diet. Post-hoc analysis showed a statistically significant increase in the levels of VLCFA that were measured in the brains of GWI mice compared to controls, which were lowered in GWI mice with OEA treatment (Fig. 4A, f = 1.0). Similarly, levels of MDA in GWI mice were elevated compared to control mice but these levels were lower in OEA treated GWI mice (Fig. 4B, f = 0.96). Several omega-3 and omega-6 FFA were altered in GWI compared to control mice on normal diet (Fig. 4C and Supplementary Figure 4). In addition, GWI mice treated with OEA had similar FFA levels compared to those observed in control mice on a normal diet, whereas levels of these FFA were elevated in control mice treated with OEA (Fig. 4A,B). Supplementary Figures 5 and 6 show that OEA treatment increased PPARα and PPARγ co-activator 1α (PGC-1α) expression, irrespective of GW chemical exposure.

### OEA reduces astroglia and microglia activation in GWI mice

**Figure 5:**
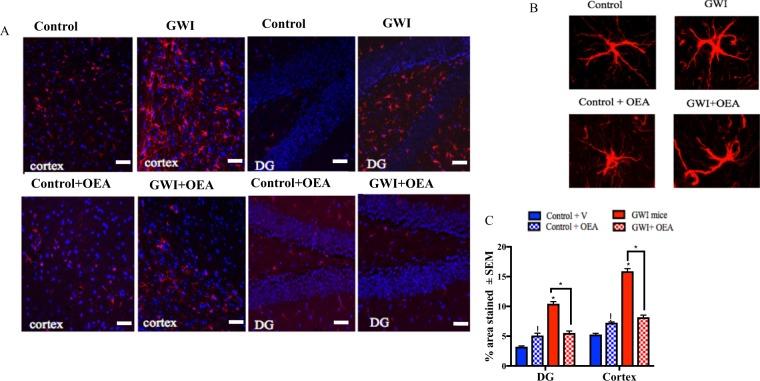
OEA treatment reduced elevated astroglia activation in GWI mice. Confocal microscopy images showing staining of astroglia with GFAP (n = 4 per group, 4 serial sections for each animal). Scale bar 50 μm. (**A**) Images show 20x GFAP staining (red) and DAPI (blue) in the cortex and the DG of mice from all 4 treatment groups. (**B**) Images show 100x magnification of GFAP stained astroglia in the cortex of each study treatment group. (**C**) Quantification of GFAP staining from 20x images. There are significant increases in GFAP staining for both the cortex and the DG within the hippocampus of GWI compared to control mice. GWI mice treated with OEA had similar levels as control mice on normal chow. Control mice treated with OEA had higher levels than control mice on the normal diet. *p ≤ 0.05.

**Figure 6:**
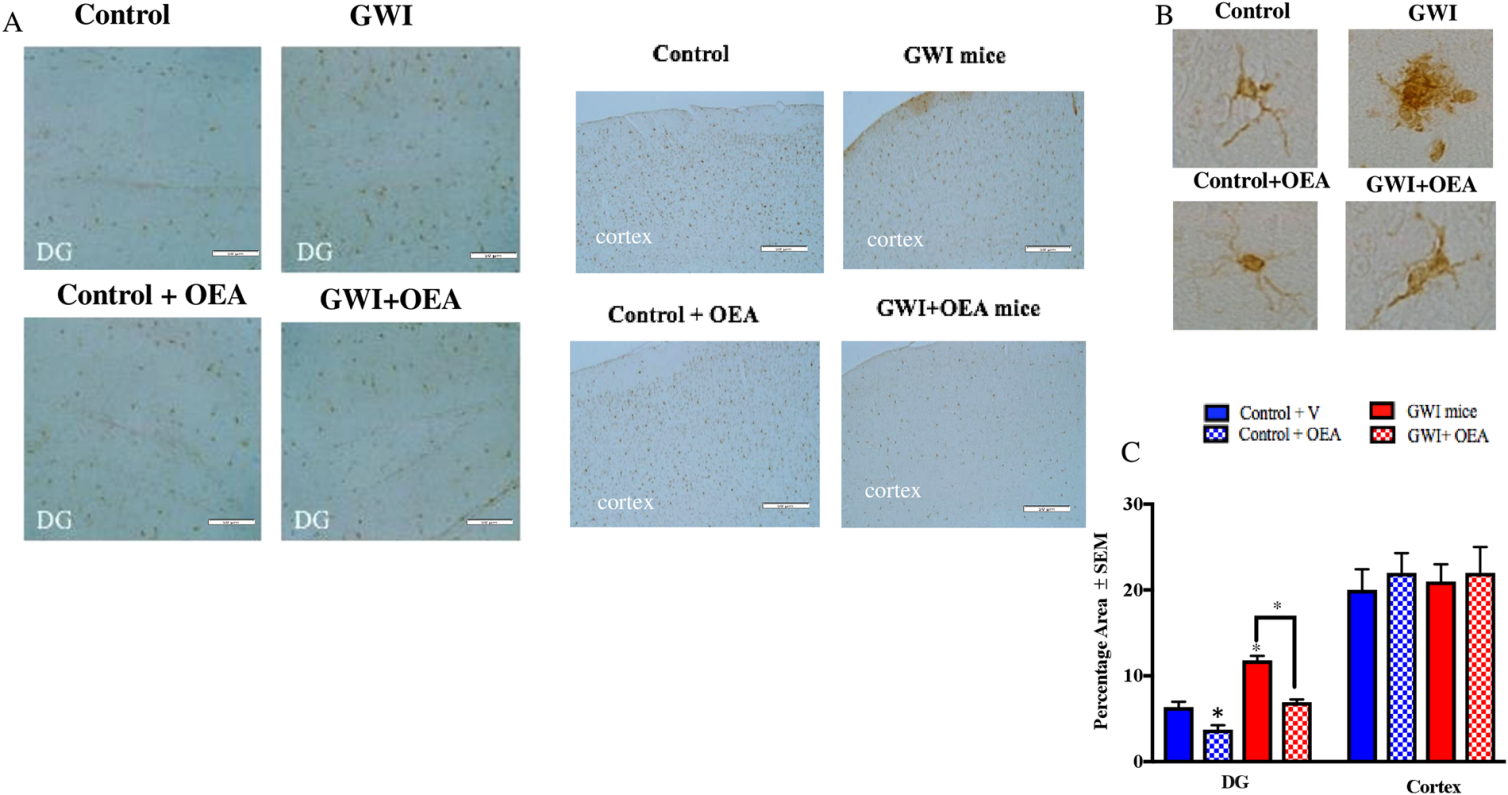
OEA treatment reduced microglia proliferation in GWI mice. Light microscopy images showing staining of microglia with IBA-1 (n = 4 per group, 4 serial section for each brain). Scale bar 50 μm. (**A**) Images show 20x Iba1 staining in the cortex and DG of mice from all 4 treatment groups. (**B**) Images show 100x magnification of Iba1 stained astroglia in the cortex of each study treatment group. (**C**) Quantification of Iba1 staining from 20x images. Levels of Iba1 staining within the DG was lower in OEA treated GWI mice compared to GWI mice on normal chow. Levels of Iba1 in GWI mice were higher in the DG compared to control mice. There were no differences in the cortices of GWI mice compared to control or OEA treated GWI mice *p ≤ 0.05.

Astroglia and microglia, which participate in protecting the brain and modulating inflammation^36–39^, are upregulated in the brains of GWI mice at long-term post-exposure time-points^8^. Using immunohistochemistry followed by confocal microscopy, an increase in the glial fibrillary acidic protein (GFAP) staining of astroglia was observed in the cerebral cortex and the dentate gyrus (DG) of GWI compared to control mice, and was significantly decreased in OEA treated GWI mice compared to GWI mice on a normal diet (p < 0.05, Fig. 5A, f = 0.89 for the hippocampus and f = 0.97 for the cortex). Immunohistochemistry staining with ionized calcium binding adaptor molecule 1 (Iba1) and CD45 staining followed by light microscopy was used to examine microglia proliferation and activation, respectively. There was an elevation of microglia staining with Iba1 in the DG of GWI compared to control mice on normal chow, whereas OEA treated GWI mice had much lower staining in the DG with Iba1 compared to GWI mice on control diet (Fig. 6A, f = 0.87). There was no detectable staining of CD45 in either control or GWI mice with or without OEA treatment, indicating a lack of microglia activation as detected by CD45 (data not shown). There was no neuronal cell death, as detected by Nissl staining (data not shown).

### OEA treatment reduces chronic brain inflammation in GWI mice

**Figure 7:**
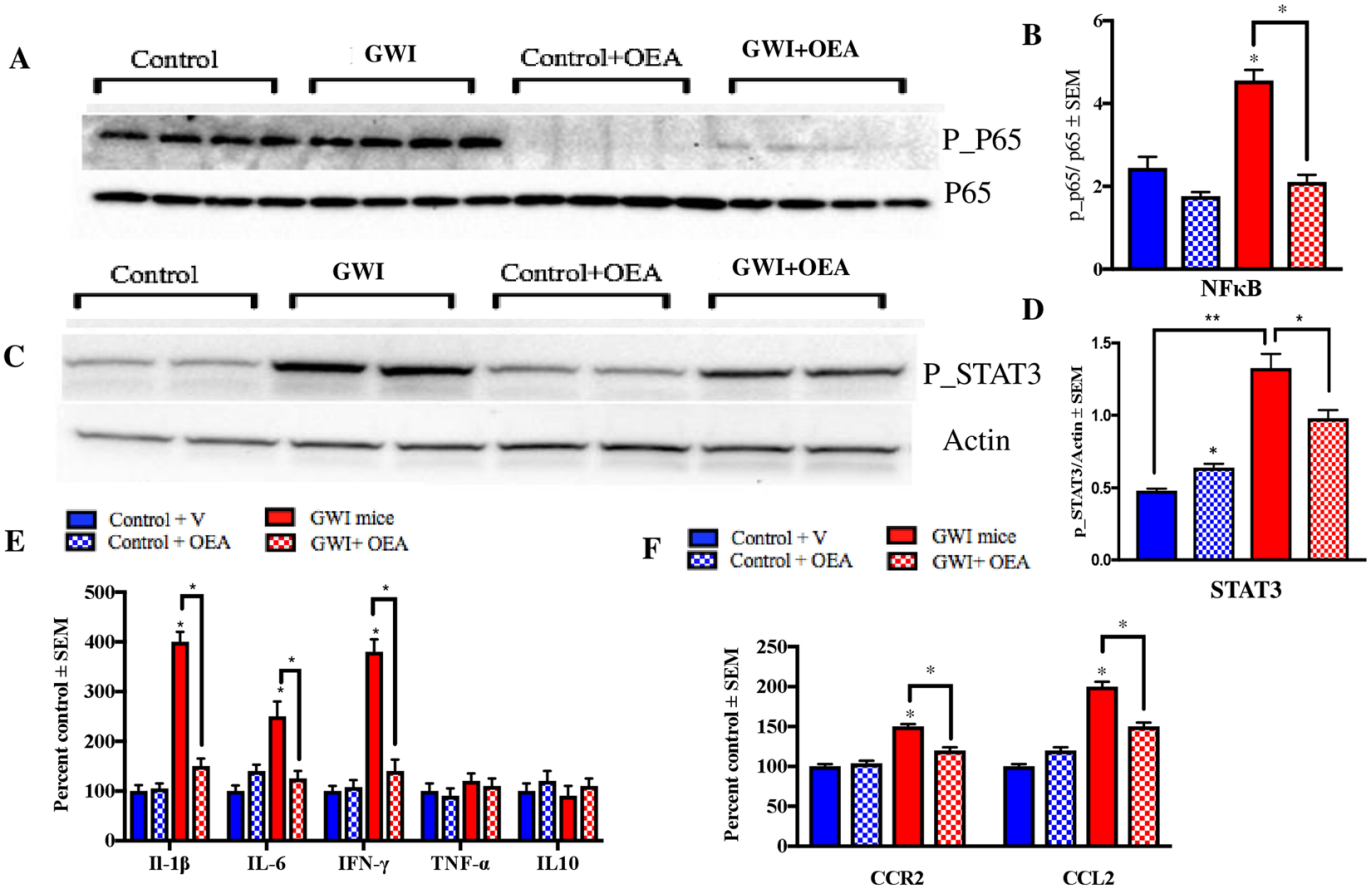
Levels of phosphorylated NFκB and STAT3 were reduced by OEA treatment in GWI mice. Mean ± SEM (shown as arbitrary units, n = 4 per group). (**A**,**B**) The ratio of p-P65/P65 was elevated in GWI mice at 11-months post-exposure. At this time-point, which corresponded with 6-months of OEA treatment, GWI mice having OEA treatment had a significantly lower ratio compared to GWI mice on normal chow. Control mice treated with OEA had non-significantly lower ratios compared to control mice on the regular diet. (**C**,**D**) Similarly, p-STAT3/Actin levels were significantly elevated in GWI compared to control mice and were also lower in OEA treated GWI mice compared to those on normal chow. Levels of STAT3 were also elevated in control mice treated with OEA compared to control mice on the normal diet. (**E**) Among the cytokines examined in the brain, IFN-γ, IL-6, and IL-1β were lower in OEA treated GWI mice compared to GWI mice on normal chow. Mice with GWI had elevated levels of these pro-inflammatory cytokines compared to control mice. These cytokines did not differ between control mice treated with OEA compared to control mice on the normal diet. (**F**) Treatment with OEA decreased the levels of CCR2 and CCL2 in the brain of GWI mice compared to GWI mice on normal chow. *p ≤ 0.05.

In order to determine if NFκB-mediated pro-inflammatory pathways could be inhibited by OEA in the brains of GWI mice, we examined the ratios of the phosphorylated p65 (p-65) to total p65 protein, which is a subunit of NFκB, phosphorylated STAT3 (p-STAT3) and several pro-inflammatory cytokines. At 11-months post-exposure to GW chemicals, corresponding to 6-months of OEA treatment, there was a significant increase in the ratios of p-p65/p65 in the brains of GWI compared to control mice, whereas OEA treated GWI mice had lower ratios compared to GWI mice on normal diet (p < 0.001, Fig. 7A,B, f = 2.79). Since p-STAT3 is thought to enhance the activation of NFκB, we examined this protein and found it to be increased in the brains of GWI mice compared to controls, and reduced in OEA treated GWI mice compared to GWI mice on normal chow (p < 0.05, Fig. 7C,D, f = 0.93). Furthermore, several pro-inflammatory cytokines, including IL-1β, IL-6, and IFN-γ were elevated in GWI mice but were significantly lower in GWI mice treated with OEA (p < 0.05, Fig. 7E, f > 3.1 for IL-1β, f = 1.6 for IL-6 and f = 0.75 for IFN-γ). These cytokines were also altered in the plasma of GWI mice and normalized in GWI mice treated with OEA (Supplementary Figure 7). Supporting cell culture studies are provided in Supplementary Figure 8.

### Chronically activated chemokine CCL2 and its receptor, CCR2, levels are reduced in OEA treated GWI mice

Chemokine receptor type 2 (CCR2) is involved in chemotaxis of myeloid cells in response to monocyte chemoattractant protein-1/chemokine (C-C motif) ligand 2 (MCP-1/CCL2)^40–42^. A chronic increase in the CCR2 and CCL2 levels was observed in GWI mice ranging from 3- to 16-months post-exposure to GW chemicals (Supplementary Figure 11). We also examined their levels in the brain at 11-months post-exposure, which corresponded with 6-months of OEA treatment. Treatment with OEA lowered both CCR2 and CCL2 levels in GWI mice compared to GWI and control mice on normal diet (Fig 7F, f = 1.49 for CCL2 and f = 0.66 for CCR2).

**Figure 8:**
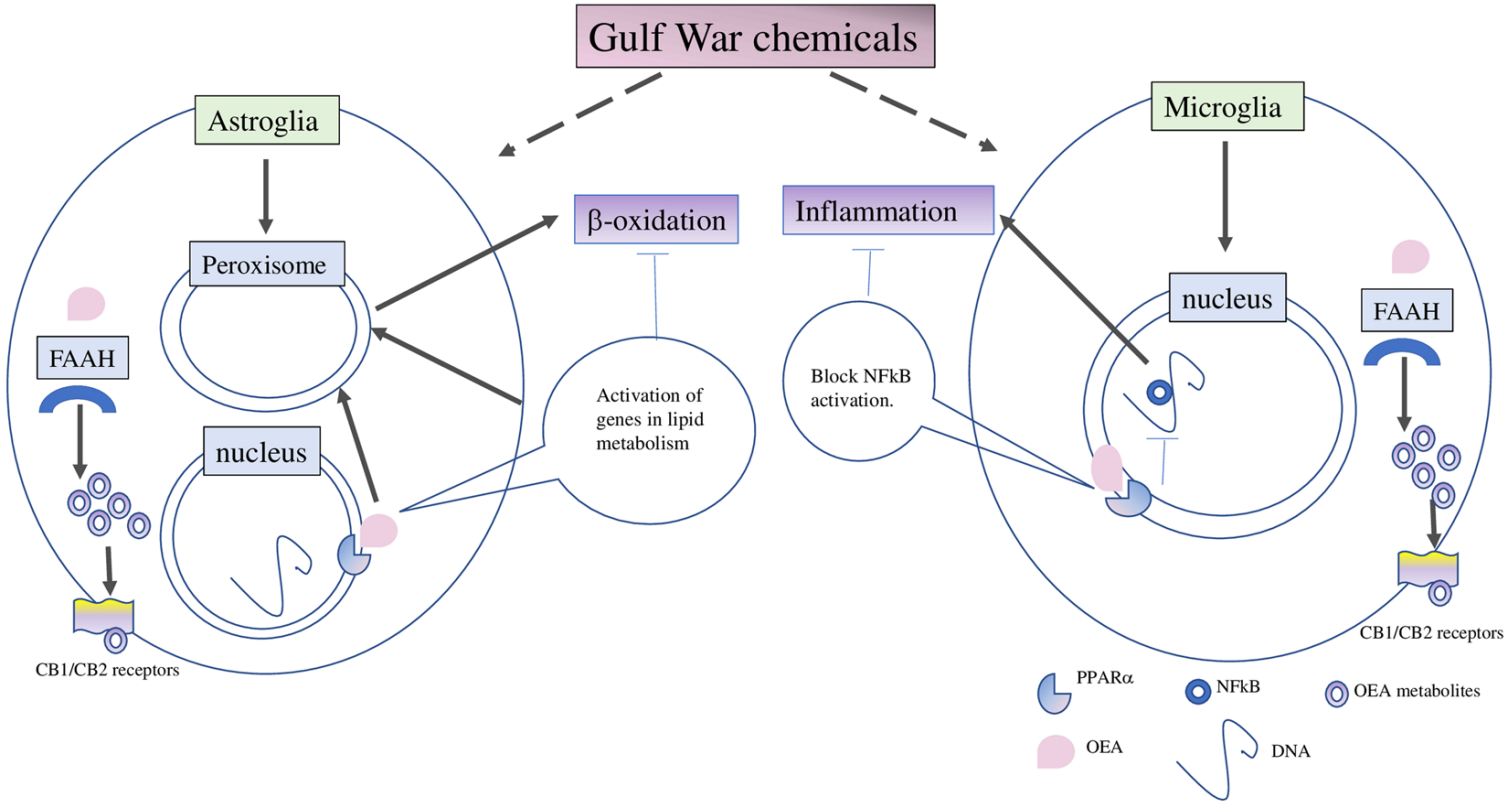
Gulf War chemicals dysregulate peroxisomal lipid metabolism in astroglia, contributing to microglia activation and inflammation which corresponds with neurobehavioral deficits. Our working hypothesis is that GW chemicals affect peroxisomal lipid metabolism in the astrocytes since astroglia activation occurs earlier than microglia activation in this GWI mouse model. We also propose that the failure of astroglia to perform its neuroprotective function contributes to the subsequent activation of microglia and neuroinflammation. Targeting peroxisomes with OEA is expected to reduce astroglia activation which corresponds with normalization of peroxisomal lipid metabolism. We also propose that OEA may also reduce NFκB activation in microglia, thereby reducing neuroinflammation. In another scenario, OEA may undergo degradation by FAAH and oxygenation by other enzymes, generating additional bioactive metabolites which may target the cannabinoid receptors and also promote anti-inflammatory responses via synergistic activation of NFκB.

## Discussion

Our exploratory studies showed that lipids which are specially metabolized in peroxisomes, including VLCFA, Pristanic acid, and DHA, were altered in plasma from veterans with GWI. These findings were supported by our previous studies showing that omega-3 DHA and ether-containing PL were disturbed in veterans with GWI^11^. Our preclinical mouse model studies also showed that VLCFA containing PL species were elevated in the brains of GWI compared to control mice^8,10^. This further supports the relevance of impaired peroxisomal β-oxidation or aberrantly active biosynthesis of these FFA to the pathogenesis of GWI (Figure 8). Currently, there are no approved therapies that can treat the underlying pathology of GWI. The present treatment strategies mostly focus on pain management with non-steroidal anti-inflammatory drugs or opioids, reducing fatigue with vitamins/antioxidants and targeting mood/cognitive issues with antidepressants. These symptom management approaches do not modify the underlying disease process associated with GWI. Hence, there remains a need for developing treatments which target the underlying pathobiology of GWI. We used OEA as a stimulator of peroxisomal β -oxidation and showed that abnormal increases in brain VLCFA, tetracosahexaenoic, and DHA levels in GWI mice were normalized after OEA treatment. These post-treatment changes also corresponded with reduced brain glia activation and inflammation and improved neurobehavioral deficits relevant to the clinical symptom presentation of GWI.

Memory impairment is one of the major complaints among veterans with GWI, and a recent meta-analysis comparing results of neuropsychological functioning in veterans with GWI across 14 studies has shown that veterans with GWI perform poorly on tasks relating to memory compared to controls^43–50^, possibly due to damage or inflammation in the hippocampal region^51–53^. Another recent study of neuropsychological functioning in military pesticide applicators from the Gulf War reported worse memory functioning in veterans exposed to pesticides and personal repellents during the war^54^. GWI animal studies have consistently shown chronic impairment in learning and memory in several rodent models of GWI^5,6,8,9,33,55^. Our current studies show that even after a couple of months of OEA treatment, GWI mice performed far better on both learning and memory-related tasks compared to all other groups, which is consistent with the currently known effects of OEA on cognition^56–58^ and may be of therapeutic value in GWI.

Other non-cognitive behavioral features were examined in this study with a particular emphasis on fatigue since clinical studies show that fatigue is one of the major symptoms of GWI, reported by nearly 79% of veterans with GWI^1,3,30,46,47^. This is supported by an imaging study showing altered axial diffusion patterns within brain areas that link cortical regions involved in pain, fatigue, and cognition in a subset of veterans with GWI^2^. At 1-month post-exposure, there was no difference between GWI and control mice on immobility during the FST, indicating no change in fatigue parameters. Interestingly, we detected increased immobility in GWI mice with repeated exposure to the FST apparatus at 3-months and a further increase at 10-months post-exposure. Interestingly, such effects were alleviated in GWI mice after 5-months of OEA supplementation. At this 10-month post-exposure timepoint, there were no indications of anxiety on the EPM and no locomotor problems or increased perimeter activity on the OFT in GWI mice. Instead, our findings on the EPM indicate disinhibition in GWI mice, evident by the fact that GWI mice were spending more time in the open arms of the EPM, which is consistent with a previous study reporting disinhibited behavior in the same model at 13-months post-exposure to GW chemicals^33^. Disinhibition is suggested to be a consequence of lesions in the hippocampus and inhibition of dorsal hippocampal functioning^59,60^. Collectively, these behavior studies suggest damage to the brain hippocampal region after GW chemical exposure and that OEA treatment could potentially alleviate these symptoms.

Consistent with previous studies^6,9,33^, we observed astroglia activation in both the cortex and hippocampus of GWI mice that was reduced by OEA treatment. As before, Iba1 staining was increased in the hippocampus of GWI mice when it was examined at 16-months post-exposure^8^. However, there was no CD45 staining in either control or GWI mice, suggesting only increases in microglia number without an overt activation. This increase was also normalized after OEA treatment in GWI mice. As can be seen, both astroglia and microglia increases appear to be key pathologies in GWI, replicated in several rodent models of GWI^6–9,33^. In fact, a role of astroglia involvement in GWI was suggested by an imaging study showing abnormal lactate utilization in subsets of GWI veterans and a blood biomarker study showing increased autoantibodies to GFAP in veterans with GWI^61,62^. While previously thought to serve in a supportive capacity within the CNS, recent studies have shown that astroglia also plays a prominent role in regulating the innate immune responses in the brain by releasing chemokines which facilitate the activation, migration, and proliferation of microglia^63,64^. Given that astroglia activation in this GWI model precedes changes in microglia, it is possible that the observed increase of microglia cells in the hippocampus may be a result of increased migration to control ongoing immune and inflammatory responses, which warrants further investigation.

While both astroglia and microglia can release CCL2, in conditions with an active immune component and an absence of obvious neuronal damage, astroglia appears to be the major producers of CCL2^65^. Our current study shows that CCL2 levels are chronically elevated in GWI compared to the control which is consistent with findings of increased mRNA of this protein in another mouse model of GWI that was exposed to an organophosphate pesticide^54^. The release of CCL2 helps recruit macrophage/microglia to the damaged area and attracts CCR2 expressing monocytes from the periphery in response to injury or chemical exposure^66,67^, contributing to inflammation via CCR2 activation through NFκB and STAT3 pathways^40,41^. Given that we do not observe an overt neuronal death in our GWI mice at any of the chronic post-exposure timepoints^6–8^, the increase in CCL2 is likely due to inflammation rather than cell death. Consistent with these studies, we also observed increases in phosphorylation of NFκB and STAT3 in the brains of GWI mice, which corresponded with an increase in several pro-inflammatory cytokines, such as IL-1β, IL-6, and IFN-γ. Increases in serum levels of these cytokines are also observed in veterans with GWI compared to control veterans^20,68,69^.

Treatment with OEA can potentially alleviate the inflammatory aspects of GWI described above. This can be supported by our studies showing that OEA exerts anti-inflammatory effects in GWI by inhibiting NFκB activity and downregulating many pro-inflammatory cytokines. However, metabolism of OEA by fatty acid amide hydrolase (FAAH) may target the brain cannabinoid receptors and some of the observed effects could be mediated by these alternative pathways and warrant an evaluation^70^. Furthermore, given this impact of OEA on the endocannabinoid system, translation of OEA treatment into humans in the context of cannabinoid use may produce some adverse effects and will require close monitoring.

One major limitation of this study is that OEA affected lipid homeostasis in control mice treated with OEA, which corresponded with lowered anxiolytic response in the EPM test and higher immobility in the FST. Despite that, we did not observe any adverse effect on memory or an increase in lipid peroxidation and inflammation in these mice. However, given the role of OEA in lipid metabolism and reducing inflammation, increasing its level in the absence of underlying inflammation or metabolic dysfunction warrants further examination. Our exploratory work into the mechanism of these effects suggests that PPARα and PGC-1α elevation could have contributed to possible increases in peroxisome proliferation and mitochondria biogenesis and that increases in lipids could reflect these changes^71–73^. We do expect that additional pharmacokinetics and pharmacodynamics studies will help identify a safe and effective dose for chronic administration in veterans with GWI.

## Conclusion

This study provides support that OEA treatment can restore normal peroxisomal lipid profiles in a well-characterized GWI mouse model, corresponding with improvement in neurobehavioral symptoms relevant to GWI. These studies also show that OEA treatment leads to a reduction of brain inflammation in this GWI mouse model, a pathology that is highly relevant to the etiology of GWI. These data provide critical support for future translational studies to find an optimal and safe dose of OEA for treating subjects with GWI.

## Future Directions

Both clinical and animal studies now point to an association between peroxisomal dysfunction and GWI and, therefore, further evaluation of the enzymatic processes and proteins involved in the biosynthesis and metabolism of VLCFA and pristanic acid is required. Studies are also required to examine the influence of genes involved in transport and metabolism of these lipids. One of these genes include the apolipoprotein E (APOE), genetic variations of which are implicated in the differential modulation of CNS vulnerability to pesticide exposure^74,75^. We and others have shown that microglia activation is associated with the chronic pathology of GWI in animal models^8,9^. As such, translational studies using microglia imaging approaches in both veterans and animal models of GWI will significantly advance our understanding of the role of microglia in GWI^76^. It would also be interesting to apply other technical approaches, such as live imaging of microglia using Translocator protein (TSPO)^77–79^ ligands or examining the presence of IL-1β on microglia, to determine the activation state of microglia^80,81^. Our studies also suggest a possible involvement of other myeloid cells in the brain that also warrants further investigation.

There are several different preclinical treatment approaches which are currently being explored that target different biological systems. Given the complexity of this illness, preclinical testing of drugs with different mechanisms of action are needed to maximize the translational potential of treating veterans with GWI. Future investigation is needed to examine a potential impact of OEA metabolism by FAAH on the observed treatment effects and OEA on neurogenesis since several studies have now shown reduced neurogenesis in GWI animal models^9,33^, though the relevance of neurogenesis in human adults remains to fully understood^82,83^.

## Methods

### Human Subjects

This study was approved by the Institutional Review Board (IRB) with all protocols conducted in accordance with relevant guidelines and regulations. Plasma samples from age- and gender-matched control veterans (n = 10) and veterans with GWI (n = 12) were compared in this study. A biorepository of plasma samples from GW veterans who previously consented to share their blood samples for future studies from the Boston Gulf War Illness Consortium (GWIC) and the Dynamic Modeling of GWI study was provided by our collaborators at the Boston University and NOVA Southeastern University sites. The GWI biorepository is approved by the institutional review boards (IRBs) at Boston University, NOVA Southeastern University and Miami Veterans Administration Medical Center (VAMC). All experiments were performed in accordance with the guidance from these oversight committees. Informed consent was obtained from all participating subjects. The GWI biorepository samples were all collected using the same written standard operating procedures for performing phlebotomy, plasma separation and aliquoting. All samples were stored at −80 °C and were not previously thawed and refrozen. The Kansas GWI criteria^75^ were used to determine cases of GWI and controls. The Kansas GWI criteria require that GW veterans show symptoms in at least 3 of 6 symptom domains (fatigue/sleep problems, pain, cognitive impairment, mood symptoms, gastrointestinal symptoms, respiratory symptoms, and skin abnormalities). Study participants were excluded if they reported being diagnosed with another medical condition that could explain their chronic health symptoms. The Kansas GWI case definition exclusions include veterans with a history of prior CNS or major psychiatric disorders that could affect cognitive function (e.g., epilepsy, stroke, brain tumor, multiple sclerosis, Parkinson’s Disease, Alzheimer’s disease, schizophrenia). Controls were deployed veterans from the 1991 GW who did not meet the Kansas GWI criteria or exclusionary criteria listed above. Subjects also completed demographic and health symptom questionnaires. The recruitment strategy for the biorepository also excludes subjects currently participating in clinical trials, to minimize interferences from the effects of these medications at the time of the blood draw. Subjects are also asked if they are on any steroid treatments and, to the best of our knowledge, subjects have reported no. However, no other medical information is currently obtained. A detailed demographic table is provided in Table 1.

**Table 1:** Demographics of the Gulf War veteran cohort.

	Control (GW veteran)	GWI
N total	10	12
Age (Mean ± SEM)	49.3 ± 2.6	46.1 ± 1.6 SE
Male (%)	100	100
**Ethnicity**		
Caucasian	5(50%)	4(28.5%)
African American	1(10%)	5(35.7%)
Hispanic	2(20%)	0
Asian	2(20%)	3(23%)

### Animal Handling

All procedures on mice were approved by Roskamp Institute’s Institutional Animal Care and Use Committee and were in compliance with the Office of Laboratory Animal Welfare and Laboratory animal care guidelines as described previously^8^. Eight-week-old male C57BL6 mice were purchased from Jackson Laboratory and allowed to acclimate to the new environment. All mice were placed on Standard rodent diet (Envigo, IN, USA) routine diet upon arrival. At 9 weeks of age (n = 48), 0.7 mg/kg of pyridostigmine bromide (PB) (Fisher Scientific) and 200 mg/kg of PER (Sigma Aldrich) in 100% Dimethyl sulfoxide (DMSO) was administered via intraperitoneal (i.p.) injection daily for 10 days, while the control group received only 100% DMSO^6,8,33^. Subsequently, mice were allowed to age for 5-months post-exposure, a time point when we have observed cognitive impairment^10,33^. Mice were again randomly assigned to either the OEA treatment or the control group. Mice in the OEA group were fed standard rodent chow (Envigo, MD, USA) containing a daily dose of 10 mg/kg OEA (based on the consumption of 5 g daily food intake) for up to 6 months, while the control group mice received standard chow only (11-months post-exposure, see Figure 2 for a timeline of the study). After 6 months of treatment, animals were euthanized for lipidomic, neuropathological, and biochemical studies.

### OEA Synthesis method

A 1-L round-bottom flask was charged with a mixture of benzene and hexanes (360 mL, 1:1, v/v) and oleic acid (25 g, 88 mmol). The above solution was cooled on an ice-bath to which oxalyl chloride (20 mL, 232 mmol) was slowly added over a period of 30 minutes via an addition funnel. This was followed by an addition of a catalytic amount of Dimethylformamide (DMF) (1 mL, 13 mmol). The above reaction mixture was removed from ice and then stirred at room temperature for 5 hrs. The solvents were then removed under reduced pressure and replaced with anhydrous Dichloromethane (DCM) (360 mL). To this solution, triethylamine (50 mL, 359 mmol) and 2-aminoethanol (22 g, 360 mmol) was added. The reaction solution was stirred overnight at room temperature and was quenched with H_2_O (360 mL). Layers were separated and then the organic layer was washed with H_2_O (360 mL × 3) and brine (360 mL) and dried with anhydrous MgSO_4_. After drying, the resulting residue was dissolved in n-hexane (500 mL) and was left in the refrigerator under −10 °C overnight. The resulting product was filtered, washed, and dried in vacuo to a constant weight as a white solid (24.5 g, 86%). ^1^H and ^13^C NMR of this product conform to literature reports.

### The Barnes Maze test

At 1 month post-treatment with OEA, the Barnes maze trial was performed to assess learning. Acquisition trials were conducted over 4 days where each trial was conducted for 3 min, and 4 trials were conducted for each mouse per day. Briefly, each mouse was placed in the middle of the maze and the trial ended when the mouse entered the escape box or after 3 min. Bright flood lamps were used as motivators for mice to enter the escape box, where they stayed for 1 min. If a mouse did not reach the target hole within 3 min, the experimenter guided it to the escape box. A probe trial was conducted 24 hrs after the last training session. The escape box was removed for the probe trials. Each mouse was placed in the middle of the maze and allowed to explore for a fixed interval of 1.5 min. Another probe trial was administered at 2 months post-treatment to assess long-term memory. The Ethovision software was used to track mouse movement, and total distance traveled by each mouse on the platform was used to calculate the path length. Animals were subsequently euthanized for neuropathological and biochemical studies.

### Forced Swim Test

Forced swim test was performed to measure fatigue-like behavior, according to the protocol published by Can and colleagues^84^ and was administered at 1- and 3-months post-exposure to GW chemicals and then subsequently at 10-months post-exposure, corresponding with 5-months after OEA treatment. Furthermore, repeated exposure to FST is used to induce fatigue in mice^85^. Mice were brought to the behavior testing room at least 30 minutes prior to the initiation of the testing in order to acclimate them to the testing conditions. The forced swim test (FST) apparatus was comprised of a cylindrical tank (30 cm height × 20 cm diameters) which was filled with warm tap water (approximately 22 °C) to a depth of 25 cm, preventing the tail and feet from reaching the bottom of the apparatus. Each animal was placed in the tank for 6 minutes, after which the mouse was removed to a warm and dry environment. Data were recorded and captured using the Ethovision XT software version 7, and latency to stop swimming and time spent being immobile was recorded as the outcome measures.

### The Elevated Plus Maze test

The EPM, used to determine anxiety, consisted of two open arms and two closed arms surrounded by high walls (40 cm) across from each other. The middle section that allows the animal to transit from arm to arm consisted of a square with dimensions of 12 × 12 cm. Mice could move freely within the maze for 5 min. Entries to each arm were recorded using the Ethovision system. Only one trial per mouse was administered. The distance traveled in the maze, the number of entries into each arm and the percentage of entries to open arms were calculated. The time spent in open and closed arms were analyzed as the outcome measures.

### The Open Field test

The Open Field Test was used to determine locomotor activity and exploration activity of the mice. Mice were placed in the middle of the 100 cm diameter arena delimited by opaque walls and allowed to explore for 10 min. Distance traveled and time spent in the center zone (60 cm diameter) versus surrounding periphery were recorded by automated video tracking (Ethovision system). After each test, mice returned to their home cages and the arena was thoroughly cleaned with 70% ethanol.

### Sample preparation

Mice were euthanized via cardiac puncture under anesthesia and all animals were transcardially perfused with PBS. Half brains (right hemisphere) and plasma were immediately frozen in liquid nitrogen and transferred to a −80 °C freezer until further use. Using a Dounce homogenizer, each brain hemisphere was homogenized in chilled lysis buffer containing protease (Roche, Indianapolis IN) and phosphatase inhibitor (Pierce, Grand Island, NY) cocktails.

### Immunohistochemistry and confocal microscopy

The left-brain hemispheres were fixed in 4% paraformaldehyde and embedded in paraffin. Sagittal sections (8 μm) were prepared and rehydrated in an ethanol gradient before the staining procedure. Glial fibrillary acid protein (GFAP; 1: 1000, Wako, Carpinteria, CA, USA) and ionized calcium binding adaptor molecule 1 (Iba1) antibodies (1: 5,000; Abcam, Cambridge, MA, USA) were used to stain astroglia and microglia, respectively. Primary antibodies were localized using respective fluorescent-labeled secondary antibodies. Slides were mounted in mounting media with DAPI (Abcam). ImageJ software was used to analyze the stained sagittal sections. The stained areas were calculated and expressed as a percentage of the field of view.

### Enzyme-Linked Immunosorbent Assay

Brain samples were homogenized using a Dounce homogenizer, in chilled lysis buffer containing protease (Roche, Indianapolis IN) and phosphatase inhibitor (Pierce, Grand Island, NY) cocktails. Enzyme-Linked Immunosorbent Assay (ELISA) kits for mouse CCR2 and CCL2 protein (LSBio, Seattle, USA) were used to study levels of these proteins in the brain. Brain homogenates were diluted 1:4 with the sample diluent provided with the kits, and all procedures were performed as per the manufacturer’s instructions. The total protein content of each sample was determined by the bicinchoninic acid (BCA) assay (ThermoFisher, Waltham, MA USA). Results were expressed in ng/mg of protein. The detection limit is typically <0.156 ng/ml and <10000 pg/ml for these ELISA kits, respectively. Intra-Assay: CV <4.6% Inter-Assay: CV <7.6% for CCR2 and Intra-Assay: CV <5.6% Inter-Assay: CV <6% for CCL2. There was no reported cross-reactivity with other proteins for the primary antibody used in these kits.

Brain cytokines, especially IFN-γ, IL-1 β, IL-10, IL-6, and TNF-α, were quantified using commercially available ELISA kits. LSBio ELISA kits were used to quantify IFN-γ (LS-F5065), IL-10(LS-F9770) and IL-6 (LS-F2478) (LSBio Seattle, USA). IL-1β and TNF-α (KMC0011 and BMS607-3) were quantified by using commercial ELISA kits (ThermoFisher, Waltham, MA USA). All procedures were performed as per the manufacturer’s instructions. Cytokine concentrations were normalized against the total protein content determined by BCA. Results were then expressed as a percentage to control. There was no reported cross-reactivity with other proteins for the primary antibody used in these kits.

### Western Blot

Following the BCA, equal amounts of protein (20 μg) from each sample were heated with Laemmli buffer containing beta-mercaptoethanol (BioRad, Hercules, CA, USA) and separated by 4–20% PAGE using a Tris-HCl buffer system and 18-well Criterion gels (Biorad) and then transferred to a polyvinylidene fluoride (PVDF) membrane (Biorad) overnight at 90 mA. The membrane was blocked for 1 hour in 5% blocking milk (BioRad, Hercules, CA, USA). Then, each membrane was individually immunoprobed with a primary antibody against p-p65 phosphorylated at Ser536 (1:1000, Cell Signaling), total p65 NFκB (1:1000, cell signaling), PPAR alpha (1:1000, cell signaling), p-STAT3 (1:1000, Cell Signaling) phosphorylated at Tyr 705, or actin (1:1000, Abcam) in blocking buffer overnight. After each primary antibody incubation, each membrane was incubated with the recommended dilution (1:5000) of corresponding horseradish peroxidase-conjugated secondary antibody (cell signaling) in blocking buffer at room temperature for 1 h. Protein bands were visualized using enhanced chemiluminescence detection reagents (Thermo Scientific, MA USA). Band intensities were analyzed using the ChemiDoc imaging system (Bio-Rad). Results were calculated using Image Lab software and normalized to the expression of actin protein in the samples.

### Multiplex Cytokine Assay

Selected cytokine levels in the plasma were analyzed using Meso Scale Discovery (MSD) 96-Well MULTI-SPOT®Ultra-Sensitive V-PLEX Proinflammatory Panel 1 mouse Kit, using electrochemiluminescence detection on an MSD Sector Imager™ 6000 with Discovery Workbench software (version 3.0.18) (MSD®, Gaithersburg, MD, USA). Cytokines were measured using the TH1/TH2 8-plex kit, which included 8 markers: IFN-γ, IL-1β, IL-2, IL-4, IL-5, IL-10, IL-13, and TNF-α. All assays were performed according to manufacturer’s instructions, in duplicates. Plasma samples were diluted 1:2 and added to the plate which contained the capture antibody immobilized on a working electrode. Following incubation for 1 hour, SULFO_TAG labeled detection antibodies were added to the wells. Finally, MSD buffer was added which developed electrochemiluminescence and the plate was loaded into an MSD instrument for reading. Data were acquired using a SECTOR S 6000 plate reader (MSD). Results were then expressed as the percentage to control.

### TBARS assay

Levels of MDA in brain homogenates were measured using a TBARS Assay Kit (Cayman Chemical, 10009055) as per the manufacturer’s protocol. At boiling temperature, oxidized lipids produce MDA which can be measured calorimetrically at 530–540 nm.

### Fatty acid analysis Assay

Total lipid extracts were prepared from brain homogenate and plasma samples by the Folch method^80^ and modified to minimize the sample volume used. Briefly, a 5 µL aliquot of internal standard containing C17 fatty acid was spiked into 10 uL of brain or plasma. Methanol (70 µL) and chloroform (120 µL) were then added. This mixture was centrifuged at 20,000 × g for 10 minutes to pellet proteins and other cell debris, and the supernatant was transferred to a clean tube. Next, 0.88% KCl was added (40 µL) at a volume of approximately 25% of the total reaction volume. This mixture was then vortexed for 1 minute and centrifuged as above to separate the phases. The upper phase was discarded and the lower phase was dried into a clean tube under vacuum. Samples were cleaned using 750 µL, PVDF, 0.2 µm centrifuge filters (Thermo Scientific) prior to analysis. Filters were conditioned prior to use by centrifuging with 1:1 chloroform: methanol (v/v) (200 µL) at 10,000 × g for 5 minutes and discarding the flow-through. Dried lipid extracts were resuspended in 1:1 chloroform: methanol (v/v) loaded onto the conditioned centrifuge filters, and centrifuged as above. Flow-through was then dried directly into an autosampler vial under vacuum and stored at −80 °C until further processing. Cleaned lipid extracts were resuspended in LC solvents (50 µL) at a ratio of 70% solvent A (27% IPA, 42% water, 21% ACN, 0.1% formic acid, 10 mM ammonium formate), and 30% solvent B (90% IPA, 10% ACN, 0.1% formic acid, 10 mM ammonium formate) for LCMS analysis of total lipids. A Thermo EASY nLC 1000 liquid chromatograph coupled with a Thermo LTQ/Orbitrap mass spectrometer with nanoflex ESI source was used for nano-HPLC-MS sample analysis. Samples were injected onto an Acclaim PepMap^TM^ 100, 75 µm × 2 cm, nanoViper, C18, 3 µm, 100A trapping column using mobile phase A as the loading solvent with the outlet flow directed to waste. Following sample loading, the outlet flow was directed to the analytical column (Acclaim PepMap^TM^ RSLC, 75 µm × 15 cm, nanoViper, C18, 2 µm, 100A) for chromatographic separation of lipid species. Gradient elution was carried out by the gradient programs shown below in Table 2. All samples were kept at 7 °C in a cooled autosampler tray for the duration of the analysis. Data was acquired by full scan MS in both positive and negative modes with a mass range of 130–2000 m/z (13 μscans/sec, spray voltage: 1500 V, resolution: 30,000, max inject time: 200msec). Tracefinder^TM^ software (Thermo Scientific) was used for peak identification and integration for lipid species in each run. Target compound lists of expected analytes for each chosen lipid class were used to find peaks of interest with ion windows of 5 ppm mass accuracy for the expected ions.

**Table 2:** Gradient Program for Positive and negative Total Lipid Runs.

Time	% Solvent A	% Solvent B	Flow Rate (nl/min)
00:00	70	30	250
01:00	50	50	250
40:00	2	98	250
50:00	2	98	250
50:01	70	30	250
65:01	70	30	250

### Statistical analyses

Post-hoc power calculations of only the primary outcomes for human studies are performed given the exploratory nature of this work. For human studies, the primary outcome of interest was peroxisomal lipids and therefore VLCFA, pristanic acid and TBARs levels were used. For the sample size used in the study, a power of >95% with the effect size d = 2.86 at α = 0.05 was detected for TBARS using the means and the standard deviations (SD) for control and GWI subjects. A power of 84% was detected for pristanic acid with a Cohen d = 1.17 at α = 0.05 whereas a power of 74% was detected with d = 1.0 at α = 0.05 for VLCFA. For behavior studies, we considered cognitive deficits as the primary outcome measure and the probe data were used for these calculations and observed a power of 65% with f = 0.43 at α = 0.05. For the FST, a power of >95% was observed with the f = 1.1 at α = 0.05. For EPM, we detected a power of >90% with the f = 0.59 at α = 0.05. Power calculations of biochemistry were restricted to VLCFA and TBARs measurements as primary outcomes of interest. For VLCFA, a power of 88% was detected with f = 1.0 at α = 0.05. For the TBARs assay, we detected a power of 79% with the f = 0.96 at α = 0.05. Both astroglia and microglia activation in the hippocampus were considered primary outcome measures for immunohistochemistry analyses. For astroglia activation, we obtained a power of 72% with f = 0.89 at α = 0.05 for the hippocampus and a power of 79% with f = 0.97 at α = 0.05 for the cortex staining. For iba1, we observed a power of 71% with f = 0.87 at α = 0.05. Data are expressed as mean ± SEM. Differences between means were assessed using one-way analysis of variance (ANOVA) or t-tests as appropriate. Neuropathological, western blot and ELISA data for protein markers described above were analyzed using ANOVA to determine statistical significance. For the lipidomics studies, a mixed linear model regression (MLM), Fisher’s least significant difference (LSD) correction, and the Benjamini–Hochberg procedure (B-H) were used for multiple-test corrections and to control the false discovery rate (FDR) for hypothesis testing of primary outcomes and as applicable. Barnes Maze data were analyzed using MLM for the acquisition trials and ANOVA for the probe trials. All other behavior tests were analyzed using ANOVA. All data were analyzed using SPSS version 22.0.0 (IBM Corporation, Armonk, NY). B-H (α = 0.05) was calculated using Excel. P < 0.05 was considered significant.

## Electronic supplementary material

Supplementary Information
