# Author Correction: Oleoylethanolamide treatment reduces neurobehavioral deficits and brain pathology in a mouse model of Gulf War Illness

**DOI:** 10.1038/s41598-019-46255-z

**Published:** 2019-07-24

**Authors:** Utsav Joshi, James E. Evans, Ross Joseph, Tanja Emmerich, Nicole Saltiel, Carlyn Lungmus, Sarah Oberlin, Heather Langlois, Joseph Ojo, Benoit Mouzon, Daniel Paris, Michael Mullan, Chao Jin, Nancy Klimas, Kimberly Sullivan, Fiona Crawford, Laila Abdullah

**Affiliations:** 1 Roskamp Institute, 2040 Whitfield Ave, Sarasota, FL 34243 USA; 2 Open University, Milton Keynes, United Kingdom; 3 James A. Haley Veterans’ Hospital, Tampa, Florida USA; 4 NOVA Southeastern University, Ft. Lauderdale, FL USA; 5 Miami VAMC, Miami, FL USA; 6 Boston University School of Public Health, Boston, MA USA

Correction to: *Scientific Reports* 10.1038/s41598-018-31242-7, published online 27 August 2018

The Acknowledgements section in this Article is incomplete.

This work is supported by two CDMRP awards (GW1300045 and GW150056) to Dr. Laila Abdullah, a VA Merit award (1I01CX000469) and a CDMRP award (GW080094) to Dr. Fiona Crawford, and by the Roskamp Foundation. Plasma samples from GW veterans were made possible through a CDMRP GWI consortium award (GW120037) to Dr. Kimberly Sullivan, and a VA merit and a CDMRP award to Dr. Nancy Klimas. We would like to thank Anastasia Edsell and Maxwell Eisenbaum for their assistance with reviewing & editing the manuscript. Disclaimer: The contents do not represent the views of the Department of Veterans Affairs or the United States Government.

should read:

This work is supported by two CDMRP awards (GW1300045 and GW150056) to Dr. Laila Abdullah, a VA Merit award (1I01CX000469) and a CDMRP award (GW080094) to Dr. Fiona Crawford, and by the Roskamp Foundation. Parts of the work were also supported by a VA Merit award (5I01RX002260) to Dr. Laila Abdullah. Plasma samples from GW veterans were made possible through a CDMRP GWI consortium award (GW120037) to Dr. Kimberly Sullivan, and a VA merit and a CDMRP award to Dr. Nancy Klimas. We would like to thank Anastasia Edsell and Maxwell Eisenbaum for their assistance with reviewing & editing the manuscript. Disclaimer: The contents do not represent the views of the Department of Veterans Affairs or the United States Government.

