# Supplementary material for: Oleoylethanolamide treatment reduces neurobehavioral deficits and brain pathology in a mouse model of Gulf War Illness
Source: Sci Rep. 2018 Aug 27;8:12921. doi: 10.1038/s41598-018-31242-7 (PMC6110778; doi:10.1038/s41598-018-31242-7)
Supplement: Supplementary file 1 — Supplementary Information [file 41598_2018_31242_MOESM1_ESM.pdf]

Oleoylethanolamide treatment reduces neurobehavioral deficits and brain pathology in a mouse model of Gulf War Illness.

Utsav Joshi[1,2,3], James E. Evans[1,3], Ross Joseph[1,3], Tanja Emmerich[1,3], Nicole Saltiel[1,3], Carlyn Lungmus[1,3], Sarah Oberlin[1], Heather Langlois[1,3], Joseph Ojo[1,2], Benoit Mouzon[1,2,3], Daniel Paris[1,2,3], Michael Mullan[1,2,3], Chao Jin[1], Nancy Klimas[4], Kimberly Sullivan[5], Fiona Crawford[1,2,3] and Laila Abdullah[1,2,3*].

[1]Roskamp Institute, 2040 Whitfield Ave, Sarasota, FL 34243

[2]Open University, Milton Keynes, United Kingdom

[3]James A. Haley Veterans' Hospital, Tampa, Florida

[4]NOVA Southeastern University, Ft. Lauderdale, FL, USA and Miami VAMC, Miami FL

[5]Boston University School of Public Health, Boston, MA, USA

Supplementary methods:

In vitro study:

Murine macrophage RAW 264.7 cells were obtained from the American Type Culture Collection (ATCC) and grown in Dulbecco's Modified Eagle Medium (DMEM) F12 medium (life science St. Louis, MO) containing 10% fetal bovine serum (FBS) and 1% mixture of antibiotics (penicillin, streptomycin sulfate) and antimycotics (amphotericin B). Cells were incubated in a humidified 5% CO_2 atmosphere at 37°C and subsequently challenged with lipopolysaccharide (LPS) from E. coli (Sigma Aldrich) at 10ug/ml to induce NFkB activation. Oleoylethanolamide (OEA), docosahexaenoyl ethanolamide (DHEA), Prostaglandin E2 ethanol amide (PGE2EA) (Cayman chemical, Ann Arbor, MI) at 10μM, 1μM, 100nM each were used to investigate possible inhibition of NFkB phosphorylation by these compounds. Cells were incubated with either DMSO alone, with LPS and DMSO, or with each test compound in combination with LPS and DMSO. Conditioned media (CM) were collected and analyzed for the quantification of cytokines. The cells were lysed with mammalian protein extraction reagent (mPER) along with protease inhibitor to quantify NFkB phosphorylation and PPAR-alpha expression in these cells.

XTT and LDH assays

Viability of RAW cells in the presence of the CM for 24 hours was assessed by the XTT (Sigma-Aldrich), and cytotoxicity was determined by the presence of lactate dehydrogenase (LDH) (Roche) according to manufacturer's instructions.

Table1. Demographics of the Gulf War veteran cohort.

	**Control (GW veteran)**	**GWI**
**N total**	8	12
**Age (Mean±SE)**	49.3 ± 2.6	46.1 ± 1.6 SE
**Male (%)**	100	100
**Ethnicity**		
Caucasian<br>African American<br>Hispanic<br>Asian	5(62.5%)<br>1(12.5%)<br>2(25%)<br>0	4(28.5%)<br>5(35.7%)<br>2(15%)<br>3(23%)

Table 2: Gradient Program for Positive and negative Total Lipid Runs

Time	% Solvent A	% Solvent B	Flow Rate (nl/min)
00:00	70	30	250
01:00	50	50	250
40:00	2	98	250
50:00	2	98	250
50:01	70	30	250
65:01	70	30	250

Supplementary 1: Fatigue-like presentation is observed in GWI-exposed mice.
(A)FST data expressed as percent control of immobile time ± SEM (n = 24 per group). Total immobile time was significantly decreased in GWI mice at 3-months post-exposure, but no effect was seen at 1-month post-exposure. (B) At 3-months post-exposure, immobility was similar between control and GWI mice for the first 2 min, but the immobility increased in GWI mice with time. $*p \leq 0.05$

Supplementary 2: There were no significant differences between any of the groups for the speed. On the EPM, there were no significant differences between any of the groups for the speed.

Supplementary 3: OEA treatment had no effect in locomotion and exploration behavior in GWI mice. On the OFT, there were no significant differences between any of the groups for the distance travelled, speed and time spend near the wall (in zone) or in the center zone among all groups. $*p \leq 0.05$

Supplementary 4: Lipid profiles from the PB+PER mouse model at the 11month-chronic post-exposure timepoint. Result expressed as percentage to control shows all Free fatty acid detected by LC-MS.

Supplementary 5: Western blot analysis of PPAR-α protein expression in the brain. There was significant increase in PPAR-α level in the mice feed with OEA compared to non OEA controls. Histogram showing relative levels of each of the PPAR-alpha protein in all four groups.

Supplementary 6: Western blot analysis of PGC-1 α protein expression in the brain. There was significant increase in PGC-1 α level in the mice feed with OEA compared to non OEA controls. Histogram showing relative levels of each of the PGC-1α protein in all four groups.

Supplementary 7: Mice with GWI had elevated levels of pro-inflammatory cytokines compared to control mice. Among the cytokines examined in the brain, IFN-γ, and IL-1β were lower in OEA treated GWI mice plasma compared to GWI mice on normal chow.

Supplementary 8: OEA inhibits pro-inflammatory cytokine production in LPS induced RAW cells. Level of IFN-γ, IL-6 and IL-1β were measured using ELISA. The data are represented as mean + SEM (n=4).

Supplementary 9: Effects of LPS and OEA PPAR-alpha expression in LPS-induced RAW macrophage cells. LPS reduces the expression of PPAR-alpha compared to control. This reduction of PPAR-alpha expression is restored by treatment with OEA.

Supplementary 10: Effects of OEA on the NFκB inflammatory signaling pathway in LPS-induced RAW cells. Modulation of NFκB activation by ethanolamides. Activation of NFκB, induced by LPS, was reduced by different ethanolamides *in vitro*. Compared to LPS alone, LPS along with OEA, PGE2EA, and DHEA significantly decreased this ratio of p-NFκB to NFκB. Cytotoxicity results showed no significant toxicity associated with ethanolamide compounds at all doses tested. *$p \leq 0.05$

Supplementary 11: CCR2 and its ligand CCL2 are chronically increased in the brains of PB+PER-exposed mice. Mean ± SEM expressed as % control of mice on normal chow (n = 5 per group). (A) CCR2 levels were increased in GWI mice at 5- through 16-months post-exposure. (B) Levels of CCL2 were also increased in GWI mice at all of the post-exposure timepoints. *$p \leq 0.05$

Supplementary 12: Levels of phosphorylated NFκB measured by ELISA were reduced by OEA treatment in GWI mice. Results expressed in Mean ± SEM (n = 4 per group). Ratio of p-NFκB(p-P65)/total NFκB(P65) was elevated in GWI mice at 11 months post-exposure. Treatment with OEA decreased the ratio of p-NFκB/total NFκB. Western blot result of NFκB and STAT3

Supplementary 13: Representation of Western blot images of NFκB(p65) and STAT3

Supplementary 14: Representation of Western blot images of PPAR-α, PGC-1 α, and Actin

Supplementary 1

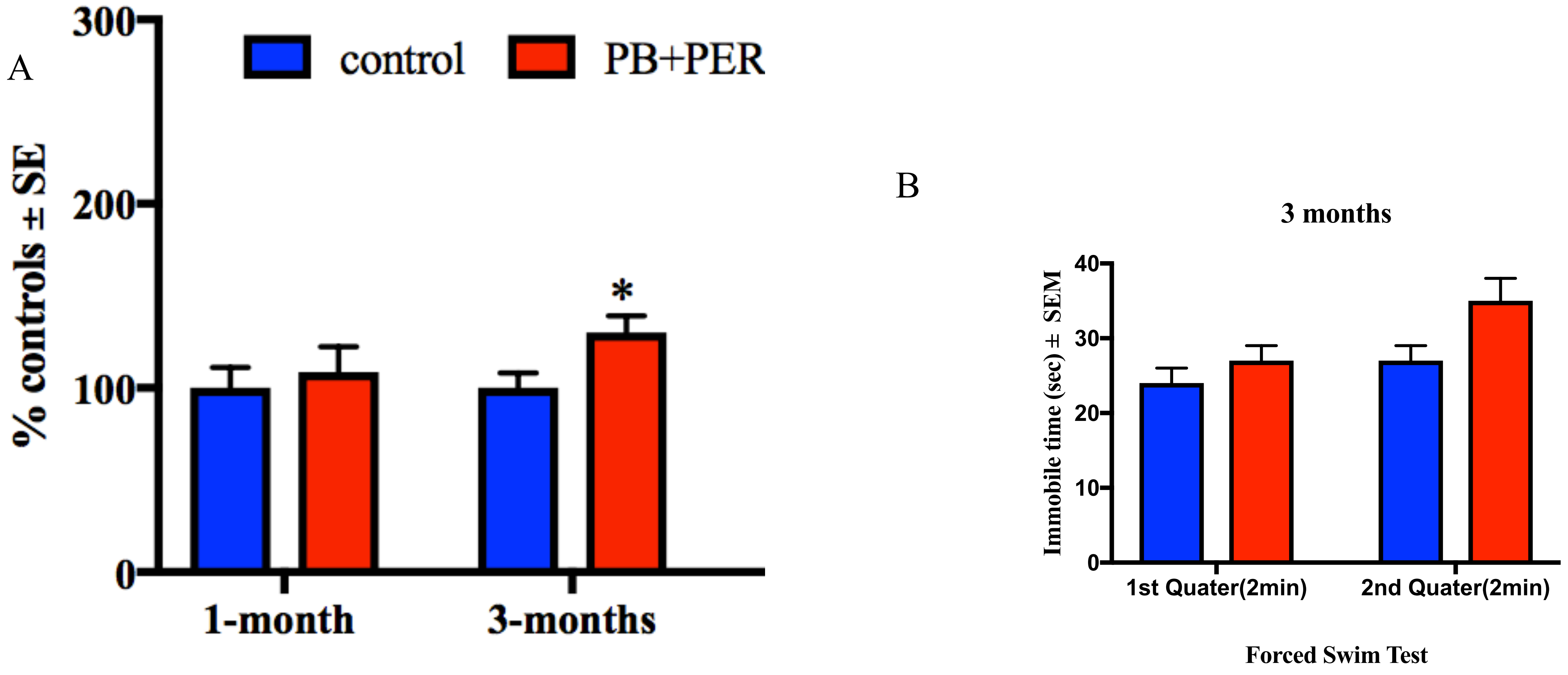

Supplementary 2

A

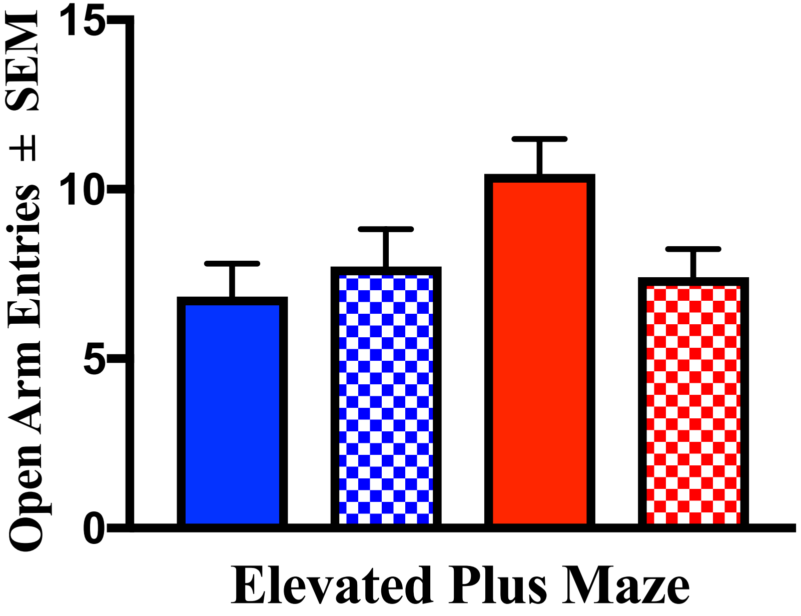

B

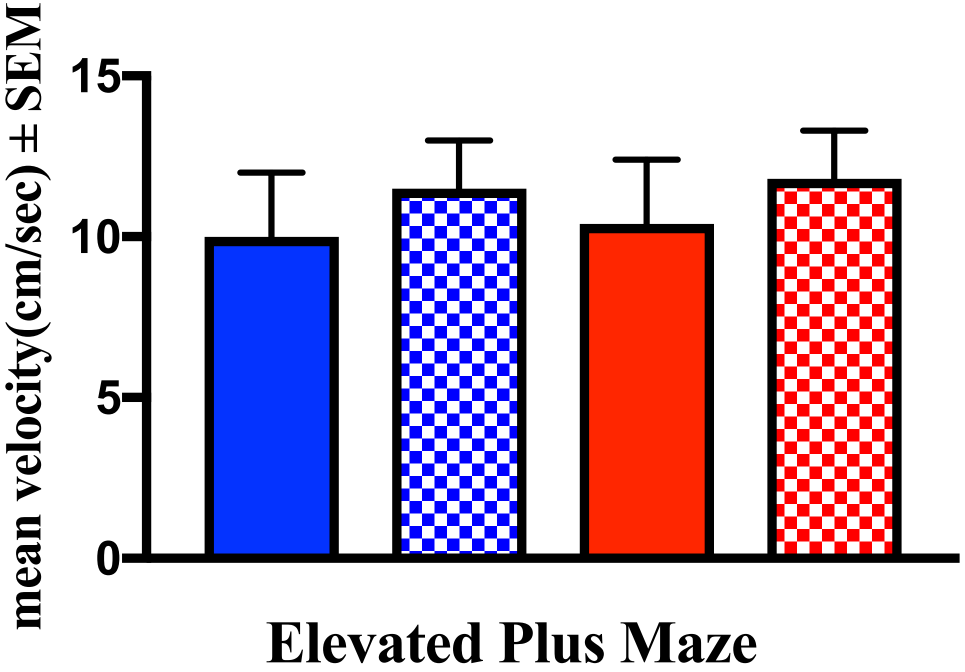

Supplementary 3

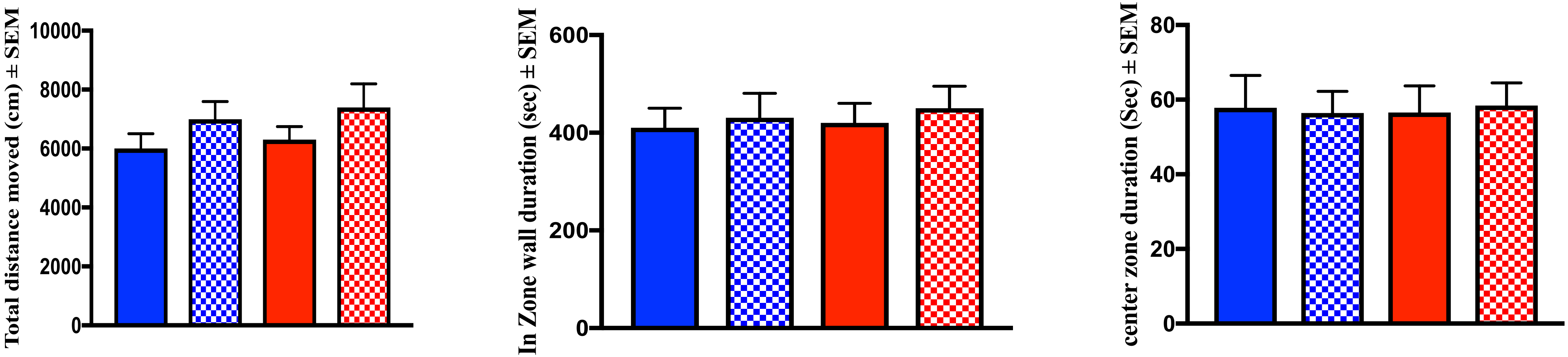

Supplementary 4

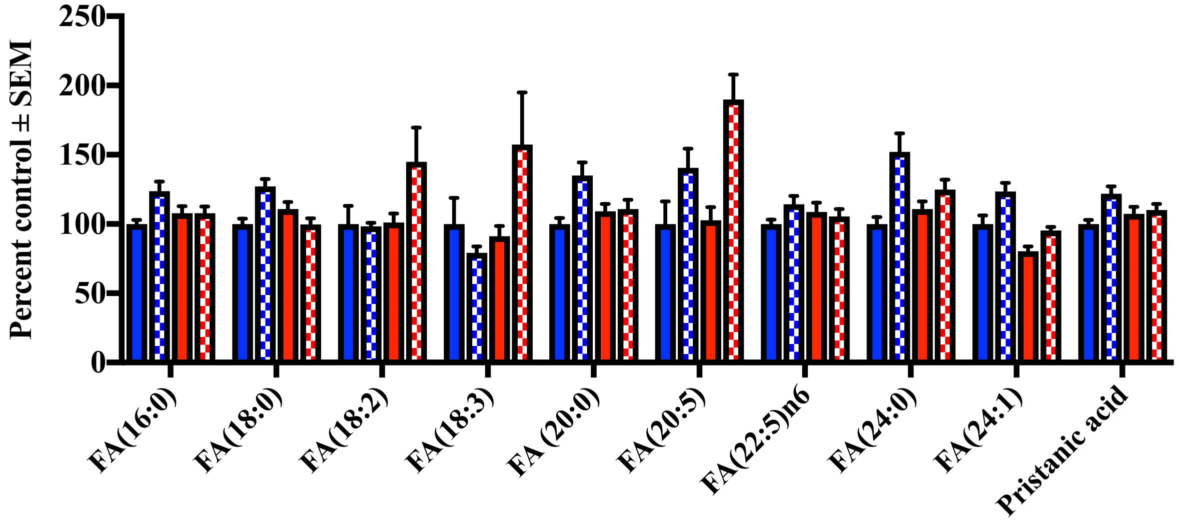

Supplementary 5

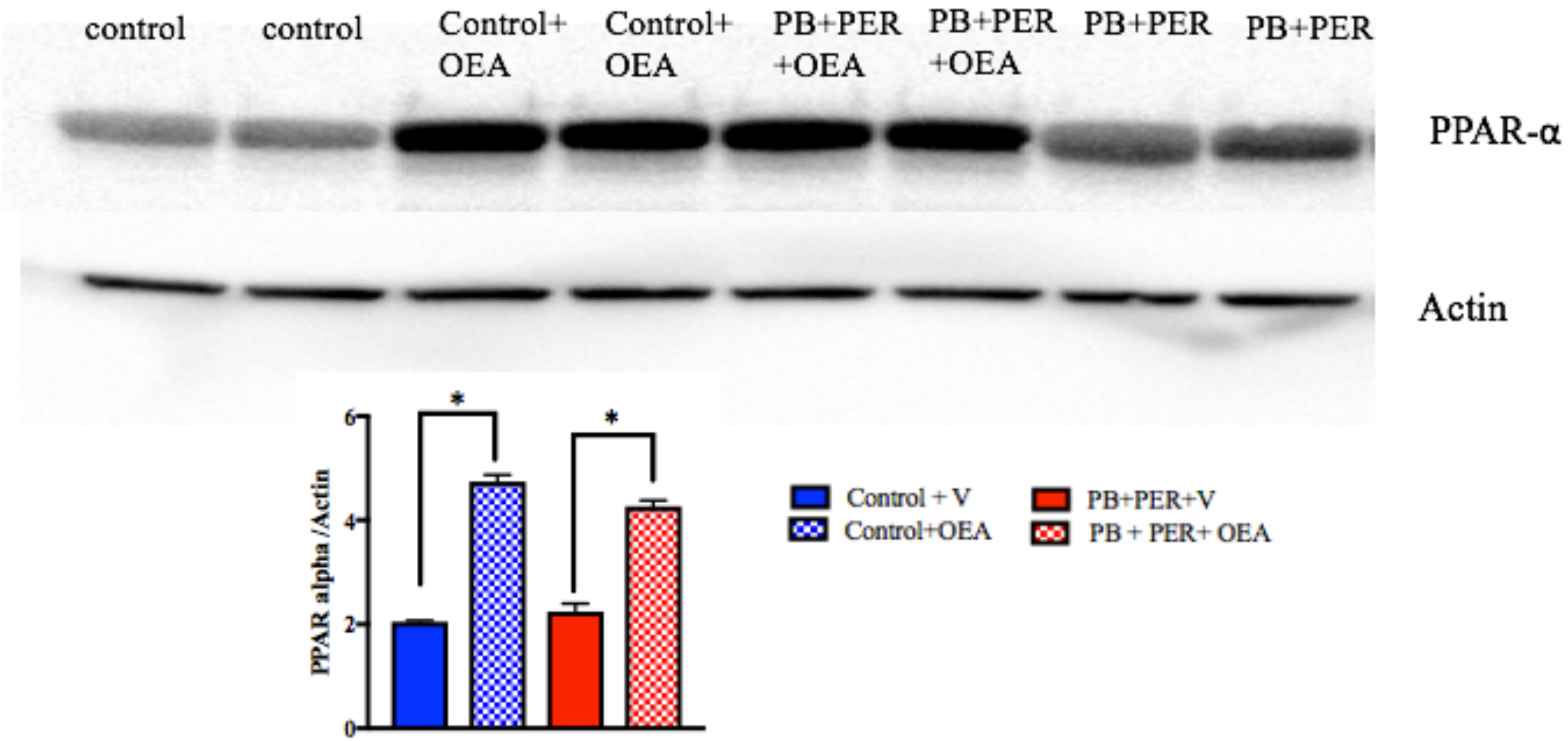

Supplementary 6

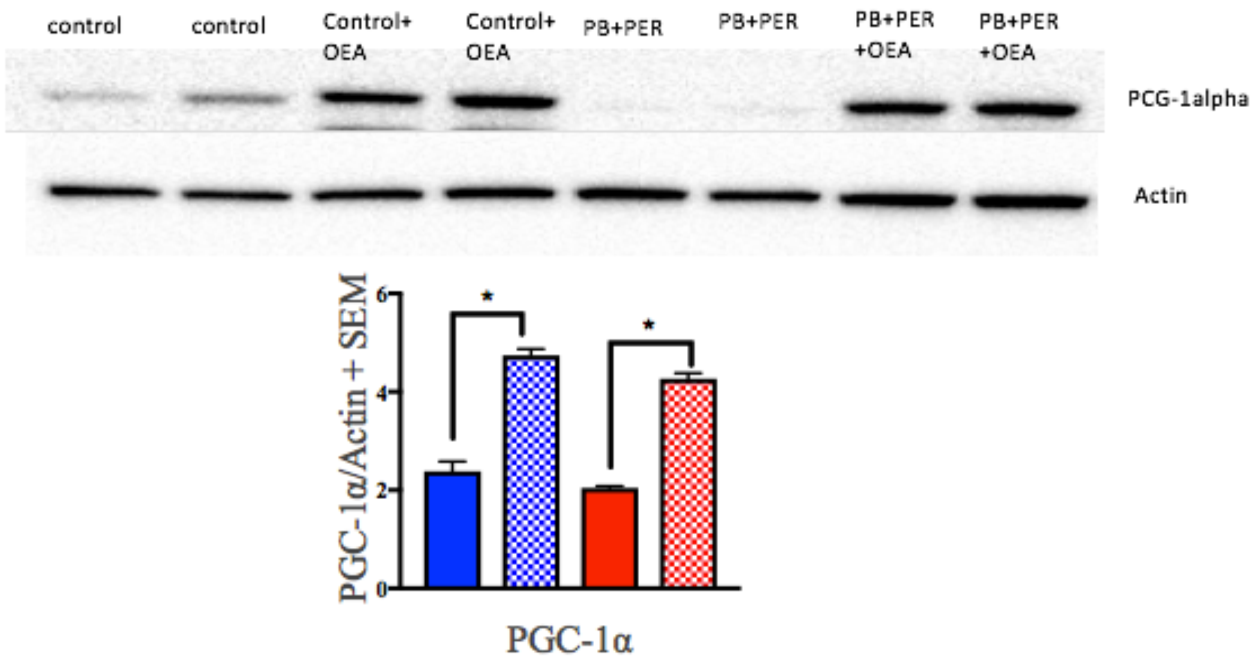

Supplementary 7

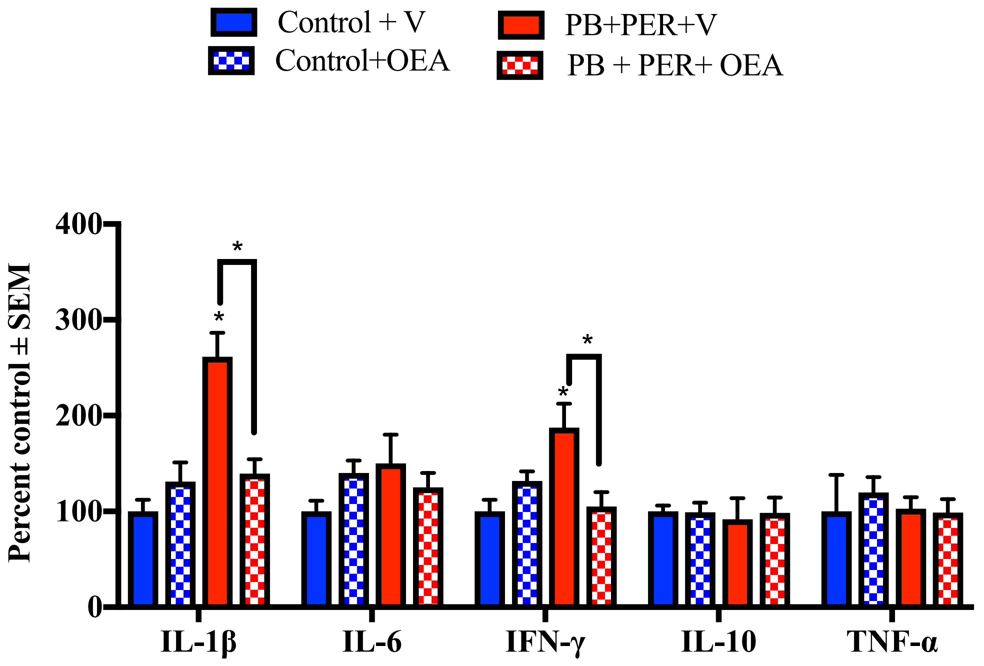

Supplementary 8

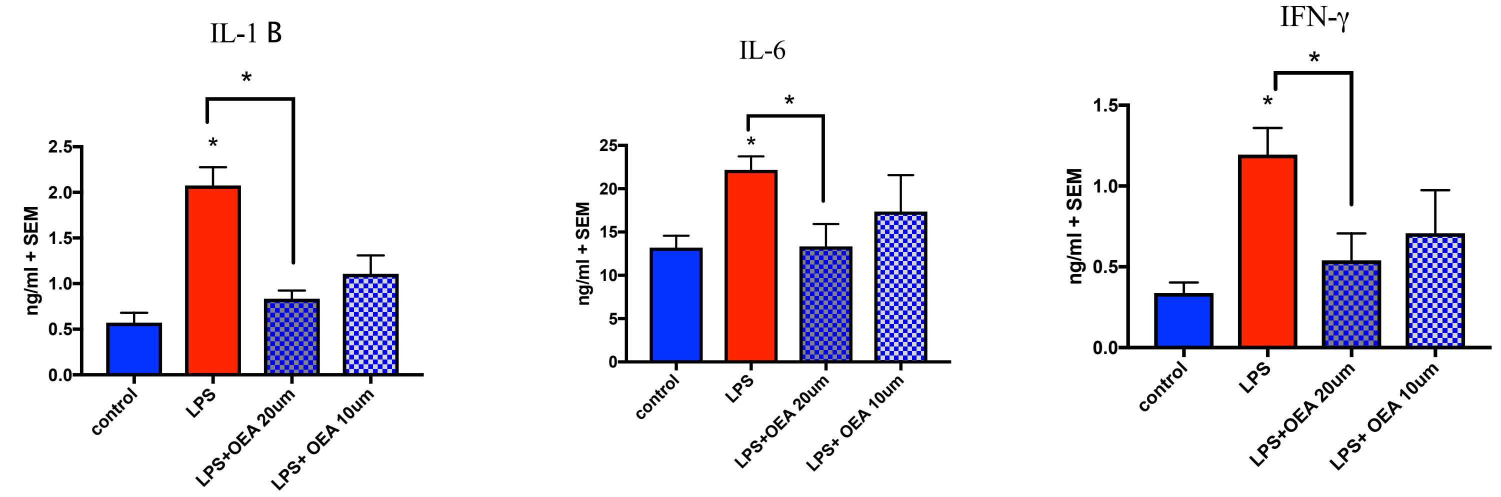

Supplementary 9

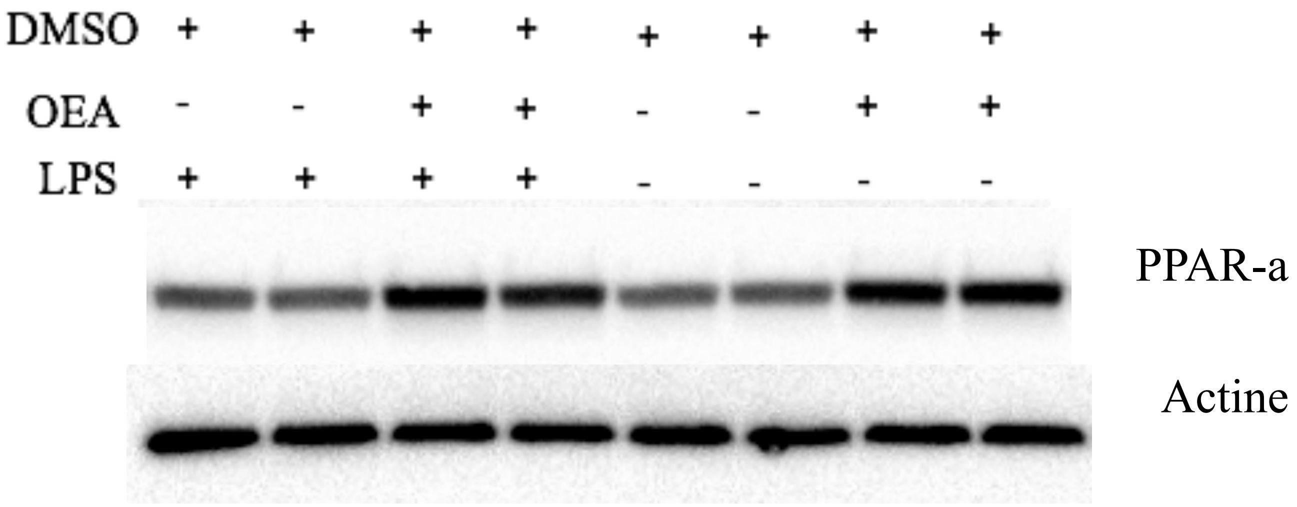

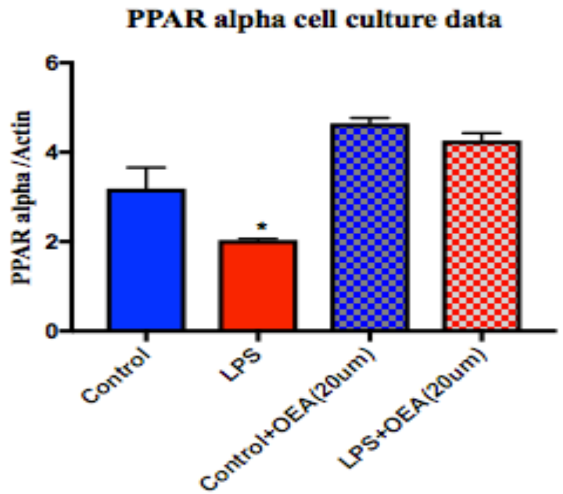

Supplementary 10

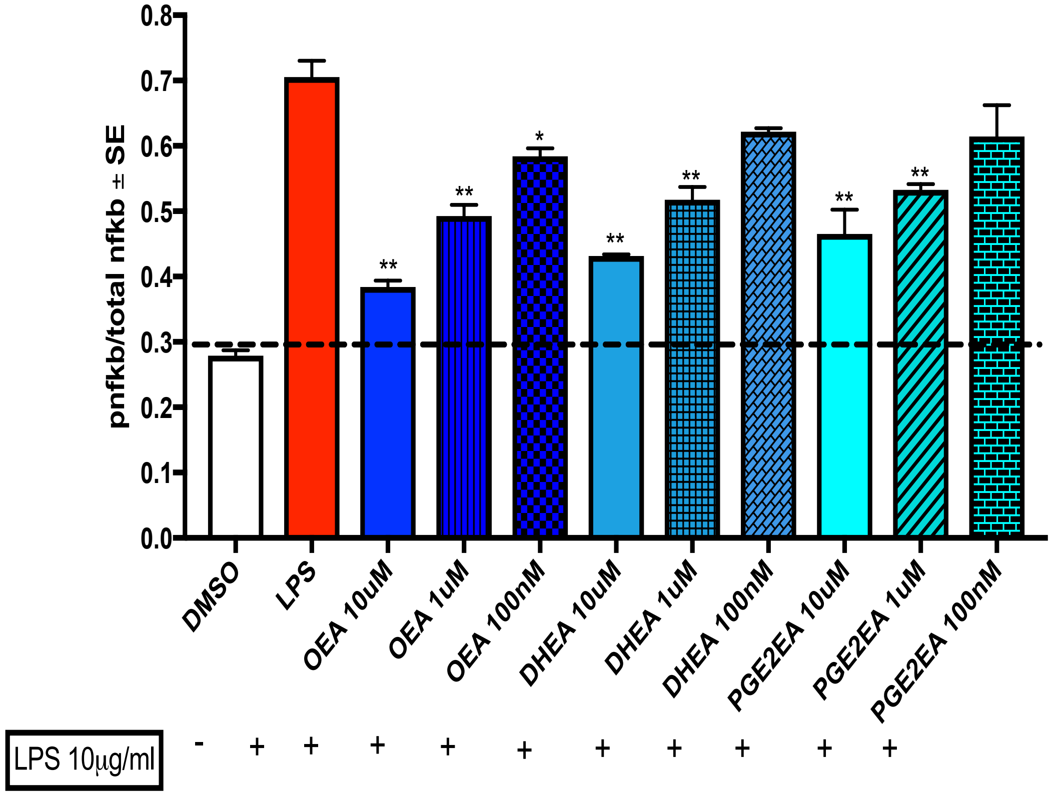

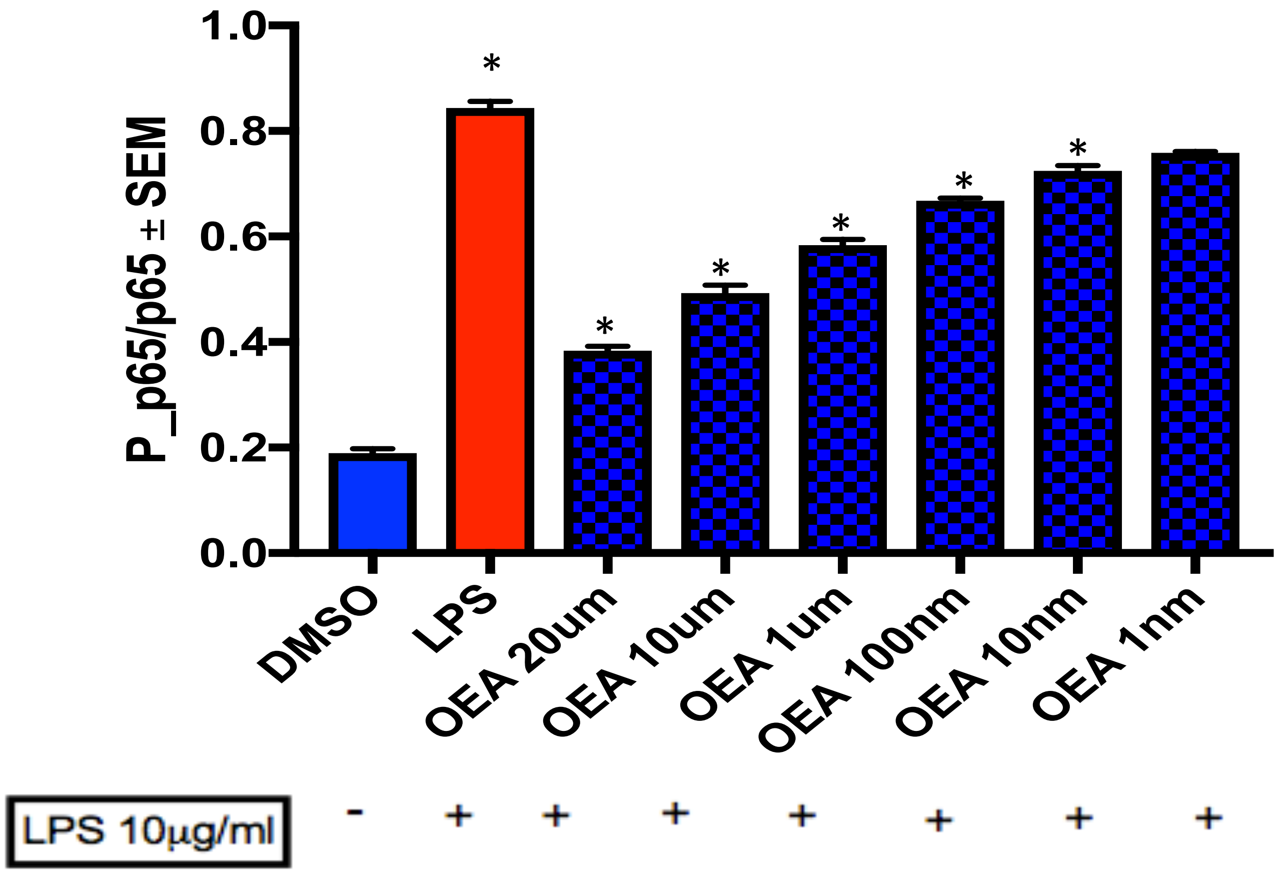

OEA= Oleoyl ethanolamide
DHEA = DHA ethanolamide
PGE2EA = prostaglandin E2 ethanolamide

Supplementary 11

CCR2

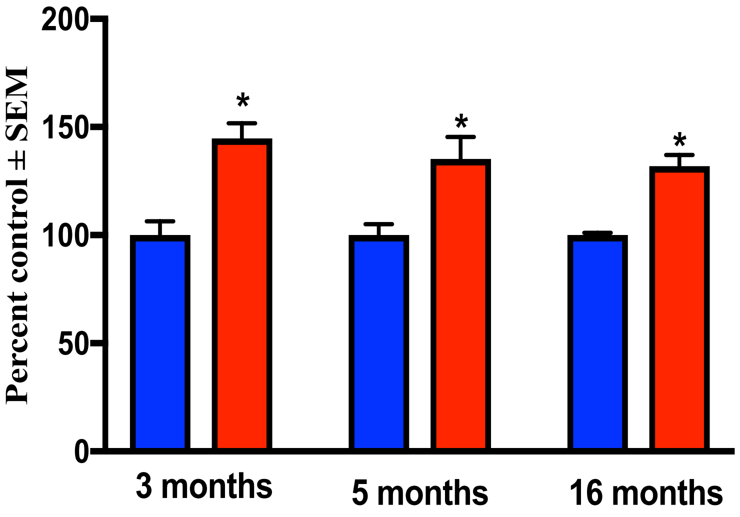

CCL2

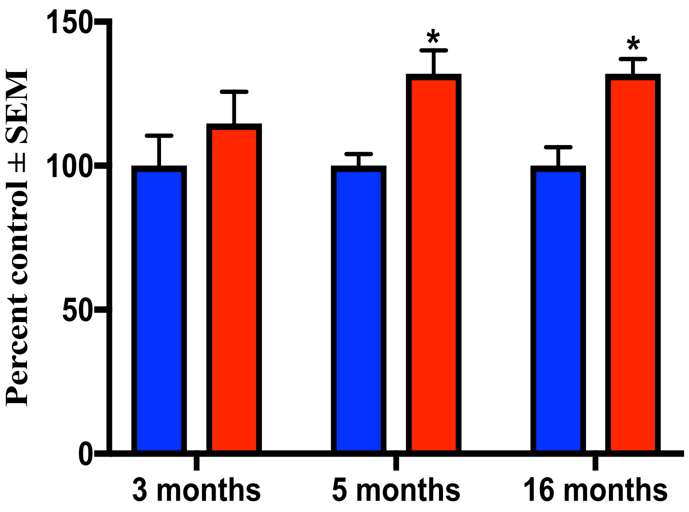

Supplementary 12

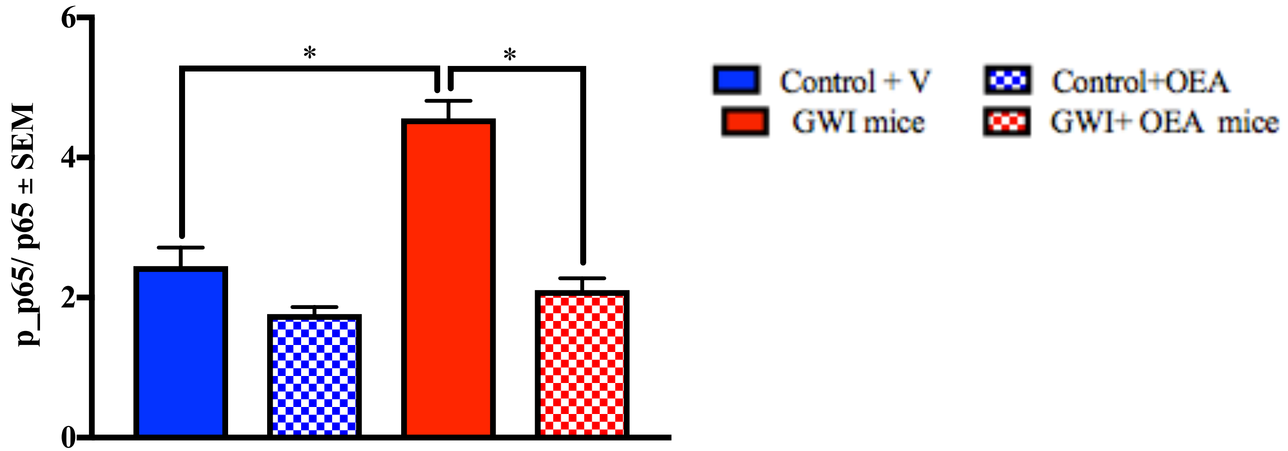

Supplementary 13

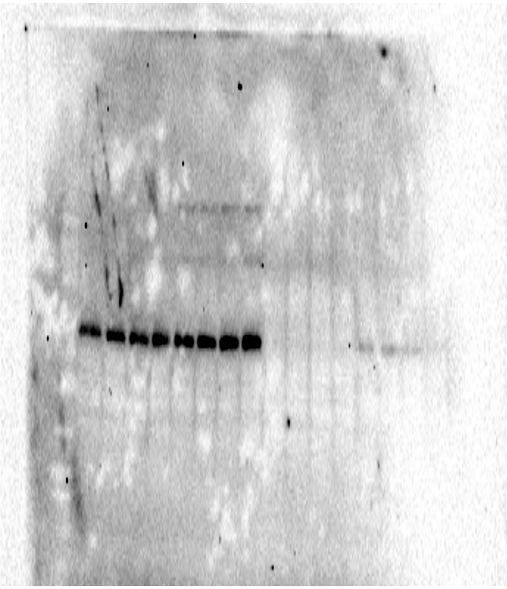

P_**NFκB**

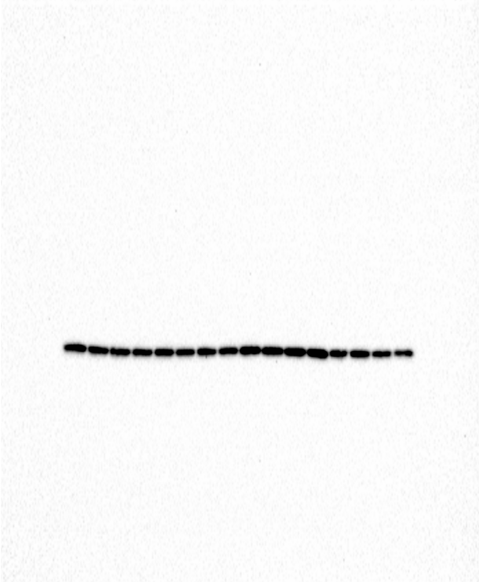

NFκB

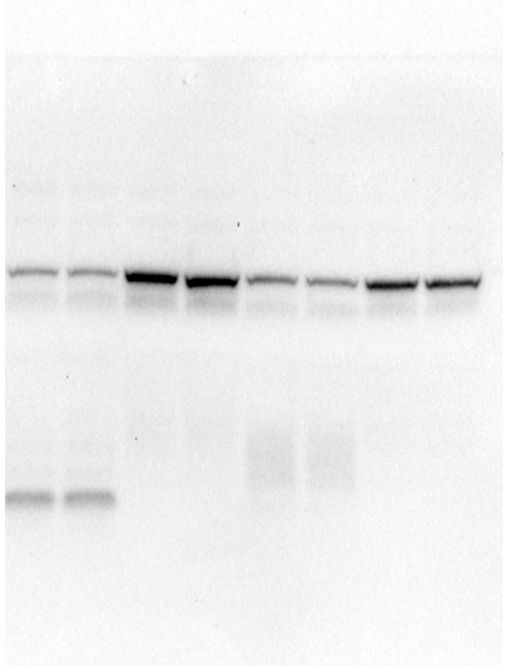

STAT3

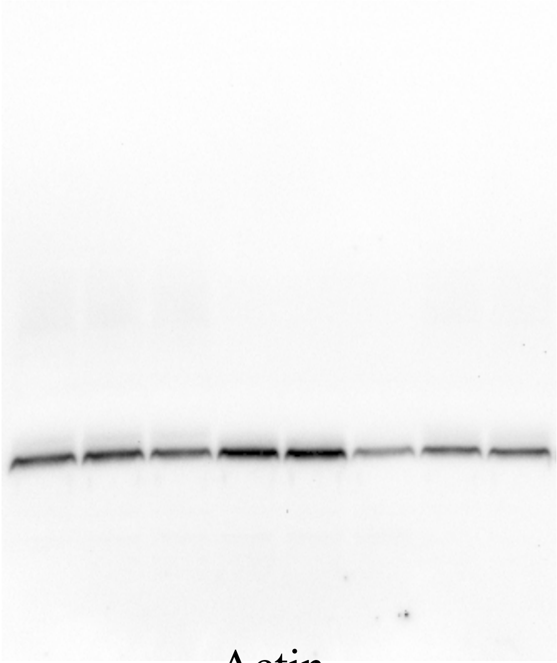

Actin

Supplementary 14

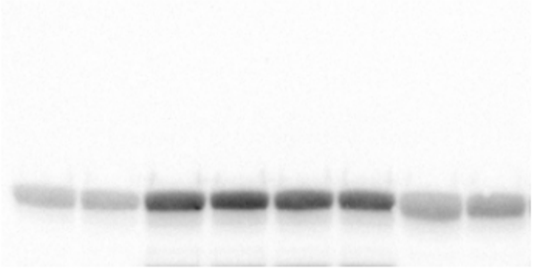

PPAR-a

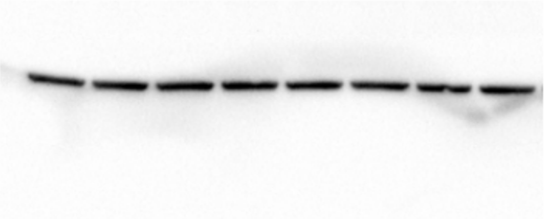

Actin

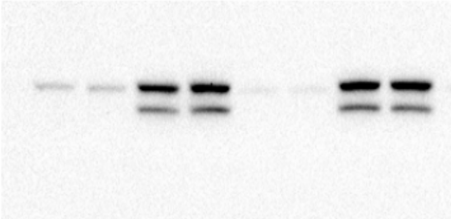

PCG-1a

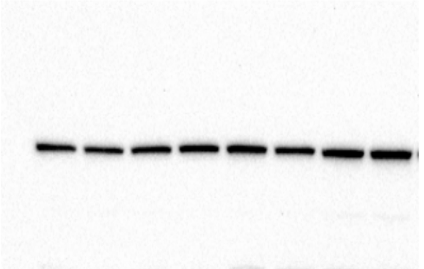

Actin

Cell culture Western blot

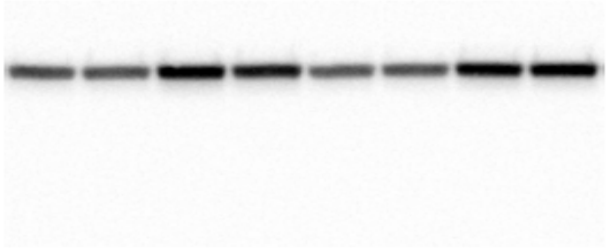

PPAR-a

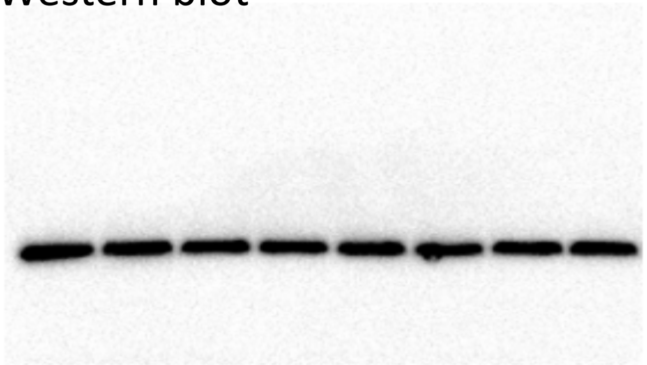

Actin
